# Antibiotic-induced gut dysbiosis elicits gut-brain axis relevant multi-omic signatures and behavioral and neuroendocrine changes in a nonhuman primate model

**DOI:** 10.1080/19490976.2024.2305476

**Published:** 2024-01-29

**Authors:** Shivdeep S. Hayer, Mackenzie Conrin, Jeffrey A. French, Andrew K. Benson, Sophie Alvarez, Kathryn Cooper, Anne Fischer, Zahraa Wajih Alsafwani, William Gasper, Mallory J. Suhr Van Haute, Haley R. Hassenstab, Shayda Azadmanesh, Missy Briardy, Skyler Gerbers, Aliyah Jabenis, Jennifer L. Thompson, Jonathan B. Clayton

**Affiliations:** a Department of Biology, University of Nebraska at Omaha, Omaha, NE, USA; b Callitrichid Research Center, University of Nebraska at Omaha, Omaha, NE, USA; c Nebraska Food for Health Center, University of Nebraska-Lincoln, Lincoln, NE, USA; d Program in Neuroscience and Behavior, University of Nebraska at Omaha, Omaha, NE, USA; e Department of Food Science and Technology, University of Nebraska-Lincoln, Lincoln, NE, USA; f Proteomics and Metabolomics Facility, Nebraska Center for Biotechnology, University of Nebraska-Lincoln, Lincoln, NE, USA; g School of Interdisciplinary Informatics, College of Information Science and Technology, University of Nebraska at Omaha, Omaha, NE, USA; h Department of Pathology and Microbiology, University of Nebraska Medical Center, Omaha, NE, USA; i Primate Microbiome Project, University of Nebraska-Lincoln, Lincoln, NE, USA

**Keywords:** Gut–microbiota–brain axis, psychiatry, metabolome, stress reactivity, microbiome-metabolome-correlation, cortisol, antimicrobials

## Abstract

Emerging evidence indicates that antibiotic-induced dysbiosis can play an etiological role in the pathogenesis of neuropsychiatric disorders. However, most of this evidence comes from rodent models. The objective of this study was to evaluate if antibiotic-induced gut dysbiosis can elicit changes in gut metabolites and behavior indicative of gut-brain axis disruption in common marmosets (*Callithrix jacchus*) – a nonhuman primate model often used to study sociability and stress. We were able to successfully induce dysbiosis in marmosets using a custom antibiotic cocktail (vancomycin, enrofloxacin and neomycin) administered orally for 28 days. This gut dysbiosis altered gut metabolite profiles, behavior, and stress reactivity. Increase in gut *Fusobacterium spp*. post-antibiotic administration was a novel dysbiotic response and has not been observed in any rodent or human studies to date. There were significant changes in concentrations of several gut metabolites which are either neurotransmitters (e.g., GABA and serotonin) or have been found to be moderators of gut-brain axis communication in rodent models (e.g., short-chain fatty acids and bile acids). There was an increase in affiliative behavior and sociability in antibiotic-administered marmosets, which might be a coping mechanism in response to gut dysbiosis-induced stress. Increase in urinary cortisol levels after multiple stressors provides more definitive proof that this model of dysbiosis may cause disrupted communication between gut and brain in common marmosets. This study is a first attempt to establish common marmosets as a novel model to study the impact of severe gut dysbiosis on gut-brain axis cross-talk and behavior.

## Introduction

Gut microbiome-mammalian cell interactions influence the development of metabolic, immune-mediated and neuropsychiatric disorders such as obesity, autoimmune disorders, autism, anxiety, and depression.^1–4^ Mechanisms by which the gut microbiome can affect central nervous system function include generation of metabolites with neuroactive properties and alteration of the hypothalamic-pituitary-adrenal axis (HPA) functioning.^4^ Gut bacteria such as *Lactobacillus* spp., *Bifidobacterium* spp., and *Escherichia* spp. secrete neuroactive substances such as serotonin, norepinephrine, gamma-aminobutyric acid (GABA) and short-chain fatty acids (SCFAs).^5–7^ The HPA axis plays an important role in stress response through the upregulation of hormones such as cortisol, and is often dysregulated in patients with psychiatric disorders.^8^ Several gut microbes have been correlated with fluctuations in levels of corticosteroids in both human^8–10^ and rodent studies.^11^ Certain probiotic bacteria, such as *Lactobacillus* spp. and *Bifidobacterium* spp., have been shown to decrease corticosteroid production in healthy humans^12^ and in mice exposed to stressful stimuli.^13^

Dysbiosis is defined as a loss of beneficial microbial organisms, the expansion of pathobionts or potentially harmful microorganisms, and/or loss of overall microbial diversity.^14,15^ While many environmental factors, such as dietary fluctuations and socio-geographical dynamics, are known to cause gut dysbiosis,^16^ oral antibiotic administration is perhaps the most well-known perturbator of microbial community structure. As evidenced in the systematic review by Zimmermann and Curtis (2019), studies demonstrating changes in gut microbiome structure and function following antibiotic exposure are numerous.^17^ Antibiotic-induced dysbiosis not only results in disruption of microbial community structure, but also alters the secretion of bacterial metabolites, including those with neuroactive properties, and thereby interferes with HPA axis regulation.^18^ Given the suspected relationships between dysbiosis and altered gut-brain communication, there is an urgent need in psychological science for the incorporation of microbiome research.^19^ Several studies in rodents have linked antibiotic-induced gut dysbiosis to the development of depressive symptomatology.^11,18^ In these studies, administration of antibiotics such as penicillins, sulfonamides, glycopeptides (specifically, vancomycin) and quinolones was shown to lead to the development of signs consistent with depression such as spending less time in open arms of elevated plus mazes, increased immobility time on forced swim and tail suspension tests, impaired object recognition, and decreased exploratory activity.^11,18^

In nonhuman primate (NHP) models, a small set of studies links gut microbiome features of dysbiosis to behavioral characteristics, however no studies have induced dysbiosis with antibiotics to assess cause–effect relationships between dysbiosis and behavioral characteristics. In a recent study using a non-rodent animal model, rhesus macaques (*Macaca mulatta*) displaying signs of depression, such as inactive huddling, reduced locomotion, and social and communicative exchanges, had lower levels of *Firmicutes* in their gut microbiomes while at the same time showing alterations in hippocampal glycerophospholipid metabolism relative to healthy controls.^20^ This provides circumstantial evidence that microbiota-gut-brain axis linkage observed in rodents may also be present in NHPs. However, additional studies using NHPs are necessary to understand microbiota-gut-brain communication, including studies that can define cause-effect relationships between dysbiosis and psychosocial behaviors and studies defining mechanisms through which aberrant signaling from dysbiotic microbiomes could mediate changes in behavioral characteristics.

To address this major research gap, we utilized a well-recognized NHP model system, the common marmoset (*Callithrix jacchus*) to determine if antibiotic-induced dysbiosis in the gut microbiome can drive specific behavioral changes and changes in metabolites relevant to gut-brain axis communication. Marmosets have a long history in neuroscience research,^21^ and are unique among many primates, as they exhibit pair-bonding, social monogamy, biparental care that is reflective of human social behavior.^22^ Further, marmosets are capable of learning complex cognitive and social tasks.^23^ The prefrontal cortex, the region of the brain responsible for regulating behavior, is also relatively similar between humans and marmosets at a structural level, making them suitable translational models to study diseases that alter behavior.^24^ There are also similarities in both gut microbiome and diet between common marmosets and humans.^25^ Hence, marmosets can serve as a translational bridge between microbiota-gut-brain-axis studies in rodents and human clinical trials.

We set out to test whether marmosets treated with antibiotics displayed signs of gut dysbiosis, as characterized through analysis of the gut microbiome, gut metabolites, endocrine signaling, and observation of behavior alterations. We specifically aimed to: i) Evaluate the characteristics of antibiotic-induced gut dysbiosis, ii) Determine whether this dysbiosis led to alterations in concentrations of fecal metabolites, especially those pertinent to gut-brain axis communication, and iii) Identify changes in behaviors and urinary cortisol concentration that may be indicative of depressive states following antibiotic administration.

## Materials and methods

### Study subjects and husbandry

Sixteen adult common marmosets (8 males and 8 females) were used in this study (mean age − 4.22 years, range − 2 to 7 years). Marmosets were housed in male-female pairs. During all phases of the study (pre-treatment, treatment and post-treatment), marmosets were provided with a consistent diet and water daily *ad libitum*. The diet was composed of a commercial marmoset diet (Zupreem, Science Diet), *Tenebrio* larvae, scrambled eggs, gum arabic (Mazuri), and a rotation of fruits and vegetables. Husbandry conditions were the same for all marmosets included in the study. Marmosets were housed in temperature and humidity-controlled conditions and maintained on a 12 h:12 h light-dark cycle. Two animal rooms in the Callitrichid Research Center (CRC) facility were used for this study. The antibiotic group and control group were housed in adjacent rooms. Female marmosets were administered birth control prior to the start of and throughout the experiment using Estrumate (cloprostenol sodium) injections, as per routine colony management practices.

### Antibiotic administration

Marmosets were divided into two groups: antibiotic group (administered antibiotics) and control group (fed vehicle, i.e., marshmallows). The antibiotic group received a once daily regimen (vancomycin = 40 mg/kg, enrofloxacin = 10 mg/kg and neomycin = 20 mg/kg) orally, using marshmallows and marshmallow fluff for 28 days. This antibiotic regimen was chosen as it targets both Gram-positive and Gram-negative intestinal bacteria, and these antibiotics are known for their minimal absorption by the gut and do not cross the blood–brain barrier.^26–28^ In terms of treatment duration, we based our approach on the range of time used in comparable rodent studies^18^ and the fact that antibiotics are often administered in complicated human infections for 2–3 weeks.^29^ Marmosets in the control group were orally administered marshmallows and marshmallow fluff in the same quantity and manner as individuals in the antibiotic group, albeit without antibiotics.

### Biological sample collection

The 56-day experiment was divided into three phases: pre-treatment (days 1–14: 14 days pre-administration of antibiotics or vehicle), treatment (days 15–42: 28 days of antibiotic or vehicle interventions) and post-treatment (days 43–56: 14 days after the end of treatment phase). Treatment phase was further divided into two phases: treatment-1 (first 14 days of antibiotic or vehicle administration) and treatment-2 (last 14 days of antibiotic or vehicle administration) for the sake of data analysis to study changes in behavior and urinary cortisol concentrations. During the pre-treatment phase, marmosets were acclimatized to experimental conditions (familiarization with research personnel, adapting to sample collection procedures, etc.). First feces and urine excreted in the morning (between 0700 and 0800 at the start of the light exposure) were collected daily during all phases of the experiment. Fecal and urine samples collected were aliquoted and stored at −80°C. Fecal samples were stored in two aliquots, one containing 95% ethanol and the other flash frozen using liquid nitrogen.

### Behavioral observations

Ten-minute behavioral observations were performed using focal sampling on marmosets 52 out of 56 days (between 1000 and 1300 h) spanning all phases of the experiment. Behavioral ethogram is described in Table 1. Missing behavioral data (4 out of 56, 7%) were imputed using the averaged values of behavior for each individual marmoset respective data for that particular experimental phase.

**Table 1.** Behavioral ethogram used in this study.

Behavior	Definition	Frequency (count)/duration
Proximity	Time spent within 10 cm of cage mate	Duration
Focal approach	Number of times marmoset under observation came within 10 cm of cage mate	Count
Focal leave	Number of times marmoset under observation moved more than 10 cm from cage mate after being in close proximity	Count
Locomotion	Time spent in movement	Duration
Scent marking	Anogenital rubbing of any surface	Count
Genital Display	Presentation of genital area whilst raising the tail	Count
Piloerection	Erection of marmoset’s hair	Count
Scratching	Self-directed scratching skin or hair	Count
Social Interaction	Positive interactions with the cage mate such as playing, sharing food, grooming or allowing to be groomed	Duration
Cage manipulation	Grabbing, pulling, or biting the cage or cage adjacent objects, such as partitions used to decrease visual access to neighboring marmosets	Count

### Gut microbiome composition

Fecal samples stored in 95% ethanol collected during pre-treatment (days l and 14), treatment (days 17, 29, 42) and post-treatment (days 50 and 56) phases were used for DNA extraction using the BioSprint 96 One-For-All Vet Kit (Indical Biosciences) using manufacturer’s instructions. Quality and concentration of extracted DNA was evaluated using Qubit fluorometer v4.0 (Invitrogen Corporation). The V4 region of the 16S rRNA gene sequences were amplified using previously described primers and protocol followed by sequencing on the Illumina MiSeq platform.^30^ QIIME2 (v2021.4) was used for denoising and quality filtering using the DADA2 pipeline. Amplicon sequence variants (ASVs) generated were aligned using MAFFT and phylogenetic trees were generated using FigTree using the QIIME2 pipeline. ASVs were annotated taxonomically using the q2-feature-classifier using the Silva database (v138) in QIIME2. Chloroplast and mitochondrial sequences were filtered and removed. ASV tables were normalized using scaling with ranked subsampling to account for the differences in the number of reads generated per sample. Normalized tables, phylogenetic trees, and taxonomy tables were exported and imported into R version 4.0.3 for downstream statistical analyses and visualization.

Shotgun metagenomic sequencing was performed on fecal samples collected during pre-treatment (day 14), treatment (days 29 and 42) and post-treatment (day 56) phases to identify species-level differences in bacterial taxa. The remaining bacterial DNA extracted for amplicon sequencing were sequenced on the Illumina NovaSeq6000 platform. Raw reads were trimmed using Trimmomatic (v0.39) (settings: SLIDINGWINDOW:4:20 MINLEN:50 LEADING:3). Trimmed reads were evaluated for quality evaluation using FastQC (v0.10) to ensure the average Phred Score was >30. Reads were then mapped to the reference marmoset genome (GCA_009663435.2 Callithrix_jacchus_cj1700_1.1) using Bowtie (v2.3.5) to remove host-associated sequences. Taxonomic profiles and relative abundances of the bacteria were estimated from the unmapped reads using MetaPhlan (v3.0.5, database-mpa_v30_CHOCOPhlAn_201901). Putative functional pathways were inferred by mapping the host-removed reads to existing pangenomes using MetaPhlan (identity threshold = 0, subject coverage = 50, query coverage = 90) and unmapped reads to the UniRef50 protein database (identity threshold = 1, subject coverage = 50, query coverage = 90) using Humann3 (version 3.6). Pathway computations were performed within Humann3 (version 3.6) using the MetaCyc database.

### Gut metabolite concentrations

Details of targeted and untargeted metabolomic runs performed to evaluate the gut metabolite concentrations are presented in Supplementary File 1. Untargeted metabolomics were performed using gas chromatography – mass spectrometry (GC-MS) methodology. The identification and quantification of the data was performed using MS-DIAL v4.48, which includes the deconvolution and alignment steps of the peaks. Two libraries were used, a local library made from running authentic standards with Kovats RI, and a public spectrum library, the curated Kovats RI with a total of 28,220 compounds (which includes the Fiehn, RIKEN and MoNA databases). The peaks were reviewed, and a final list of compounds with RI similarities >95% were reported. The peak area was normalized based on the internal standard spiked in the samples during extraction and using LOWESS (locally weighted scatterplot smoothing) for QC-batch normalization.

Two targeted metabolomic assays, SCFAs, and bile acids, were performed using GC-MS and liquid chromatography – mass spectrometry (LC-MS) methodology, respectively. Generated data was analyzed using Agilent MassHunter Quantitative Analysis and Analyst software (version 1.6.3) for SCFA and bile acid assays, respectively. For quantification, an external standard curve was prepared using a series of standard samples containing different concentrations of SCFAs and fixed concentration of the D3-acetate internal standard. The following SCFAs were assayed: acetic acid, butyric acid, propionic acid, valeric acid, and isovaleric acid. Absolute quantification of the bile acids was done similarly using an external standard curve of known concentrations of standards and internal standards. Bile acids assayed included α-muricholic acid (αMA), β-muricholic acid (βMA), chenodeoxycholic acid (CDCA), cholic acid (CA), deoxycholic acid (DCA), glycochenodeoxylic acid (GCDA), glycocholic acid (GCA), glycodeoxycholic acid (GDCA), glycolithocholic acid (GLCA), hyocholic acid (HCA), lithocholic acid (LCA), ω-muricholic acid (ωMA), taurochenodeoxycholic acid (TCDCA), taurocholic acid (TCA), taurodeoxycholic acid (TDCA) and taurolithocholic acid (TLCA). βMA, ωMA, HCA, LCA, and GLCA were removed from further analyses because these compounds were detected in less than 40% of the samples. We also defined two major bile acid pools as follows: primary bile acids (GCA, GCDCA, TCA, TCDCA, CA, CDCA) and secondary bile acids (GDCA, TDCA, DCA, LCA).

### Exposure to stressor

Excreted cortisol concentrations were measured via an enzyme immunoassay that has been validated for use in marmosets.^22^ Urine samples were diluted in distilled water (1:3,200 or 1:6,400) to fall within the standard curve and assayed in duplicate. Cortisol standards ranged from 1000 to 7.8 pg/well. The mass of cortisol is expressed in mg of cortisol per mg of creatinine (Cr) to control for variation in fluid intake and output. Creatinine was measured using a standard Jaffé reaction colorimetric assay.^31^ The intra-assay coefficient of variation (CV) for samples was 5.6%, and the interassay CVs for high and low concentration pools were 13.2% and 14.3%, respectively.

In addition to urine and feces, blood samples were collected from a subset of the study subjects during the treatment and post-treatment phase. While blood samples were collected for another unrelated study, the excessive but transient handling of marmosets during the process of net capture in the homecage, manual restraint, and subsequent induction of anesthesia (inhalant isoflurane) most likely led to additional stress experienced by these subjects.^22^ This provided us with an opportunity to study “stress reactivity” using urinary cortisol measurements (i.e., changes in urinary cortisol concentrations on the day of and before excessive handling versus the concentrations on the subsequent morning). We had 28 pairs of such urine samples (i.e., urine samples collected on the day of and next day after excessive handling) from 7 different marmosets (16 from antibiotic group, 12 from control group). We contrasted the “stress reactivity” in both groups by evaluating the pairwise differences (before and morning after exposure to stress) in urinary cortisol concentrations (see statistical analyses below).

### Statistical analyses

Bacterial taxons present in less than 10% of the samples were filtered from further analysis. Metrics of alpha diversity (including Shannon and Simpson diversity metrics) and beta diversity analysis (including Bray-Curtis and Aitchison distances) were estimated at genus level (16s rRNA data) and species level (shotgun metagenomic data) using packages “*microViz*”, “*microbiome*” and “*phyloseq*” in R. Mixed-effects general linear regression models were used to evaluate the changes in alpha diversity metrics over time in R using packages *lme4* and *lmertest*: alpha diversity ~ gender + group (antibiotics or control) + time-point (discrete) + (group x time-point) + (1|marmoset), with (group x time-point) interaction being the main outcome of interest. Day 14 was chosen as the reference time-point since that was the last day before antibiotics were administered (i.e., gut dysbiosis was induced). The results were confirmed by also making generalized estimating equation models (which do not assume the distribution characteristics of the data) using the same formula as used for the mixed-effects models above (package *geepack* in R). Principal coordinates analysis was performed and plotted on Bray-Curtis and Aicthison distances for each timepoint to evaluate visual differences in the beta diversity of the groups. Dissimilarities in beta diversity were statistically evaluated using the PERMANOVA test (100 permutations). Finally, linear discriminant analysis (LDA) analysis was performed to identify differentially abundant phyla, genera, species and predicted metabolic pathways using default settings. Since the results of Shannon index were similar to Simpson index and the results of analyses based on Bray-Curtis dissimilarity were similar to Aitchison dissimilarity, the results based on Shannon index and Bray-Curtis dissimilarity only are discussed.

Changes in behavior, concentrations of urinary cortisol and fecal metabolites (SCFAs, bile acids and relative concentrations of select untargeted metabolites) were evaluated using mixed-effects and GEE models as described above with slight variations. Zero-inflated negative binomial regression models were additionally built for rare behaviors because they had a different distribution than regularly occurring behaviors (package *glmmTMB* in R). A separate model was created for each behavior evaluated. Behaviors and urinary cortisol measurements were pooled and averaged for each phase (pre-treatment, treatment-1, treatment-2 and post-treatment) and the pre-treatment phase was used as a reference. Treatment-1 and treatment-2 sub-phases were created for behavior and urinary cortisol because data on these was collected nearly daily and data was sufficient to evaluate these parameters at a finer time-scale. Values of SCFAs, bile acids, and untargeted metabolites were transformed using log10 transformation to normalize their distributions before fitting the models and the pre-treatment phase was used as a reference point. Differences in stress reactivity between groups was statistically tested using repeated measures ANOVA (package *lme4* in R). If group-phase interaction was significant, then regression models were built *post-hoc* using phase and gender as fixed effect and marmosets as random effect for the antibiotic or control group separately to evaluate within group changes using Tukey’s test (package *emmeans* in R). If there was a significant difference between the groups during pre-treatment phase for a variable (metabolite or behavior), then the baseline values (i.e., values during pre-treatment phase) for that variable were added as an independent variable in the mixed effects models.^32^

Additionally, the results of the untargeted metabolite profiling were uploaded to the MetaboAnalyst 5.0 web interface and principal component analysis and pathway analysis was performed and plotted after log10 transformation and pareto scaling for each phase. Differentially abundant metabolites were identified by t-tests. Pathway enrichment analysis was performed by first mapping the metabolites from the untargeted run to the human KEGG database and then performing a global statistical test on the metabolic pathways detected.

Correlations between concentrations of metabolites (after log10 transformation), abundance of bacterial families (after centered log-ratio transformation using package *Tjazi* in R) and behaviors were estimated using Pearson correlation coefficient (package *Hmisc* in R). The following planned correlation tests were separately performed: 1. Between behaviors and concentrations of all the metabolites tested (fecal SCFA, bile acids, and untargeted metabolites), 2. Between relative abundance of bacterial genera based on 16s rRNA sequencing and metabolites tested, 3. Between relative abundance of bacterial genera based on 16s rRNA sequencing and behaviors tested, 4. Between abundance of metabolic pathways predicted by shotgun metagenomics and behaviors, 5. Between behaviors and urinary cortisol levels, and 6. Between urinary cortisol and bacterial genera. Correlations were considered to be significant if p-values after correcting for multiple comparisons were less than 0.05. Correlation network was plotted using package *ggnet2* in R. The p-values were adjusted for multiple comparison testing using false discovery rate correction. In this study, p-values less than 0.05 were considered as significant. All the major bioinformatic programs used are cited in Supplementary File 2.

## Results

### Antibiotics induced severe changes in gut microbiome composition

**Figure 1.**
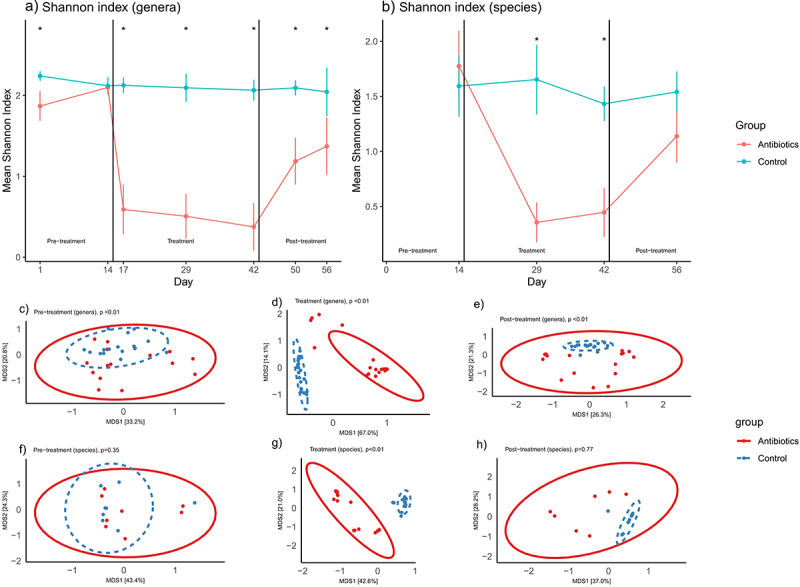
Changes in fecal microbial diversity over time. Figures 1a-b represent changes in Shannon index (alpha diversity) at the bacterial genera and species levels over time (mean ±95% confidence interval). Figures 1c-h represent changes in beta diversity (bray-curtis dissimilarity) at the bacterial genera level (c-e) and species level (f-h) over time. Bacterial genera and species were classified based on 16S rRNA sequencing and shotgun metagenomic sequencing, respectively. * signifies that the Shannon indices of the two groups were significantly different at that time-point (t-test, *p* < .05). p-values in figures 1c-h represent the results of PERMANOVA (100 permutations) at the particular phase of the experiment.

At the start of pre-treatment phase (day 1 of 56), 16S rRNA sequence data revealed significant differences in alpha diversity (Shannon index) between the antibiotic and control groups, but these differences were not present on day 14 (i.e., before the start of the interventions) (Figure 1a). Antibiotic treatment led to a statistically significant decrease in alpha diversity (Figure 1a) which persisted through the end of experiment (Figure 1a). Analysis of shotgun sequencing data revealed no significant differences in bacterial species-level alpha diversity between the groups in the pre-treatment phase (Figure 1b) but a significant decrease in this diversity was seen in the treatment phase (i.e., post-antibiotic administration) (Figure 1b), and, by the end of the experiment (post-treatment phase), partial recovery in diversity was observed (Figure 1b).

Beta diversity analyses, specifically PERMANOVA analysis of Bray-Curtis distances, revealed significant differences in population structure of bacterial genera during the pre-treatment phase (Figure 1c). The differences in these measures were significant on day 1 but not on day 14. Day 14 was the last day of the pre-treatment phase, thus prior to the start of treatment, antibiotic and control groups were not statistically different in terms of their beta diversities (Supplementary File 3). Distinct clustering and statistically significant Bray-Curtis dissimilarity were estimated between the groups after the interventions during both treatment (Figure 1d) and post-treatment phase (Figure 1e). Based on shotgun sequencing, distinct clustering, and statistically significant Bray-Curtis dissimilarity were estimated between the groups at a bacterial species level only after the interventions during treatment phase (Figures 1f–h).

**Figure 2.**
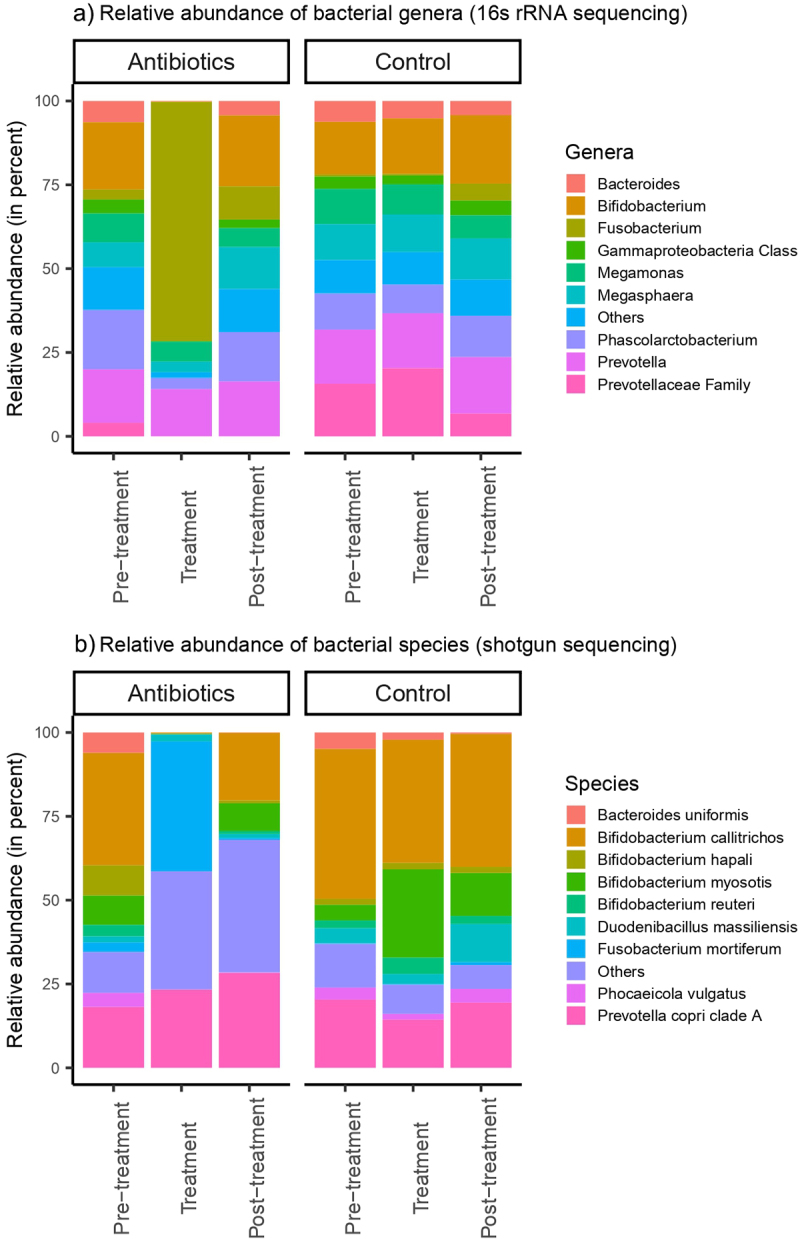
Relative abundances of a) bacterial genera and b) bacterial species in feces. Bacterial genera and species were classified based on 16S rRNA sequencing and shotgun metagenomic sequencing, respectively.

Treatment with antibiotics induced significant dysbiosis in the gut of common marmosets at both the genus-level (based on 16S rRNA sequencing) and species-level (based on shotgun metagenome sequencing). The relative abundances of the bacterial genera and species at each study phase were determined and plotted (Figure 2). The three most prevalent bacterial genera averaged across both groups in the pre-treatment phase were *Bifidobacterium* spp., *Prevotella* spp. and *Phascolarctobacterium* spp., with these three genera contributing to an average of 48.1% of the gut microbiota for each individual animal (Figure 2a). Species-level analysis, obtained by assembling and annotating shotgun metagenomic data showed similar results with *Bifidobacterium callitrichos* (39.2%) and *Prevotella copri* (19.3%) being the most abundant bacterial species present in pre-treatment phase (Figure 2b).

**Figure 3.**
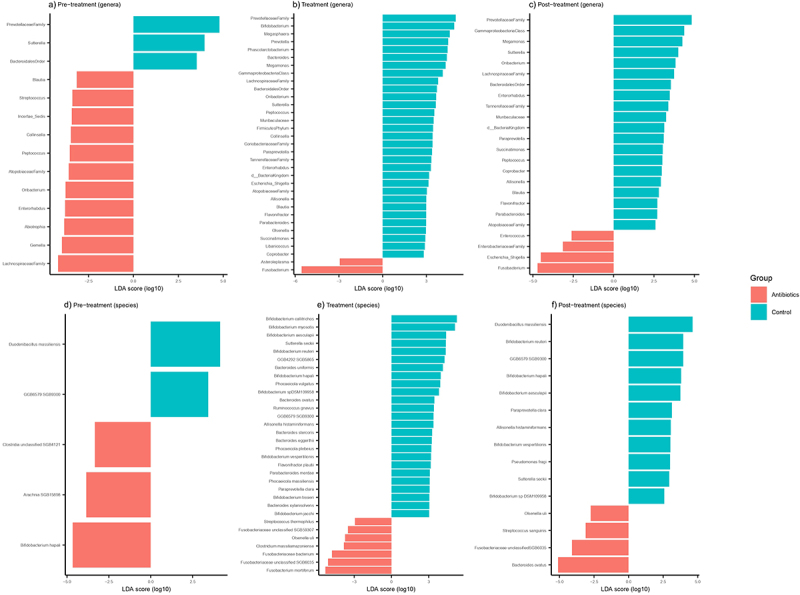
Differentially abundant fecal bacterial genera (a-c) and species (d-f) across different phases of the experiment based on LefSe analysis. Bacterial genera and species were classified based on 16S rRNA sequencing and shotgun metagenomic sequencing, respectively.

We assessed the taxa from the 16S rRNA data at the genus level showing significant treatment effects of antibiotics using LefSe, which identified 14 bacterial genera that were differentially abundant between antibiotic and control groups during pre-treatment phase (Figure 3a). There were 10 bacterial genera that were differentially abundant between the groups at day 1, but only three of these bacterial genera remained differentially abundant by day 14 (Supplementary File 3). In the treatment phase, relative to the control group, 33 bacterial genera were differentially abundant in the antibiotic group, most notable being increased *Fusobacterium* spp. abundance and decreased abundance of 31 genera (Figure 3b). Alterations in gut microbiome persisted into and throughout the post-treatment phase, with 24 bacterial genera remaining differentially abundant between the groups (Figure 3c). Based on shotgun metagenomics, five bacterial species were differentially abundant between the groups in the pre-treatment phase (Figure 3d) with this number increasing to 32 and 15 in the treatment and post-treatment phases, respectively (Figures 3e, f). The most notable taxonomic change was an increase in the abundance of *Fusobacteriaceae* (especially *Fusobacterium mortiferum*) after antibiotic treatment.

### Significant changes in behavior post-antibiotic administration

**Figure 4.**
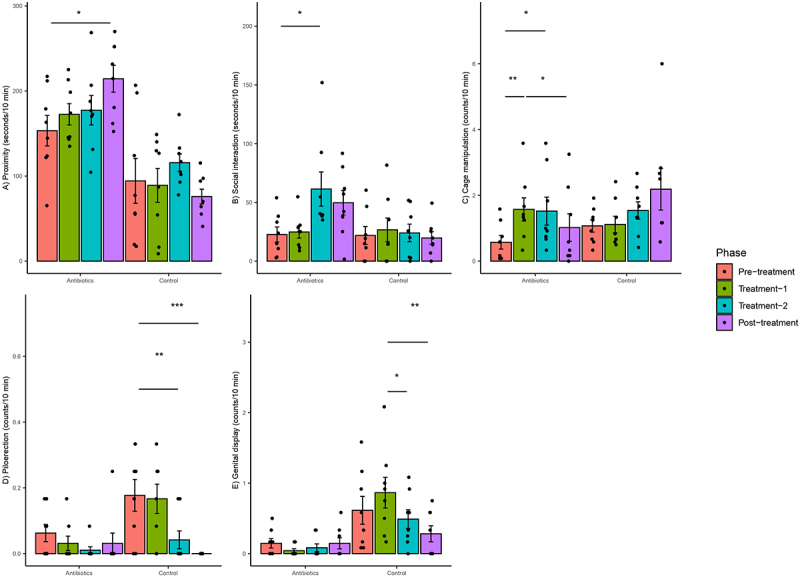
Within-group changes in behaviors (a-e) of the common marmosets during the course of the experiment. Bar-plots represent mean ± standard error. p-values highlighted were estimated from mixed effects general linear models built at the group level. *- p-value <.05, **- p-value <.01, ***- p-value <.001. Piloerection and genital display did not change significantly in zero-inflated negative binomial regression models.

Antibiotic treatment was also significantly associated with altered behavior of marmosets. There was a significant group-phase interaction for changes in five behaviors (Supplementary File 4). Most notably, there was a significant increase in cage manipulations, social interaction and proximity to partner over time in the antibiotic group as compared to the control group during treatment-1, treatment-2 and post-treatment phases relative to pre-treatment phase, respectively (Supplementary File 4). Post-hoc analysis at the within-group level confirmed that there were significant increases in these behaviors only in the antibiotic group but not in the control group (Figure 4).

There were significant group–phase interactions with respect to the changes in genital display and piloerection behaviors when counts were used in linear regression models (Supplementary File 4), but post-hoc analysis revealed that these statistical interactions were due to changes in behavior in the control group rather than the antibiotic group (Figure 4). Specifically, there was a significant decrease in piloerection and genital display in the control group across different phases (Figure 4). Models accounting for differences in the pre-treatment phase were also built for genital display, proximity, and piloerection, but the results of these models were similar to the models built without baselines values as covariates. However, the data were heavily zero-inflated because there were no observations of piloerection and genital display in 62.5 and 40.6% of the biweekly aggregated behavioral data. Hence, zero-inflated negative binomial regression models were built for these behaviors. The results were not significant for piloerection. There was a significant group–phase interaction for genital display, but these differences were not significant on post-hoc testing. The model fits of the zero inflated models than linear models were objectively better based on the distribution of residuals. Finally, there were no significant changes in the counts or durations of focal approach, focal leave, locomotion, scent marking and scratching.

### Antibiotics altered the functional pathways in the gut as predicted by shotgun metagenomics

**Figure 5.**
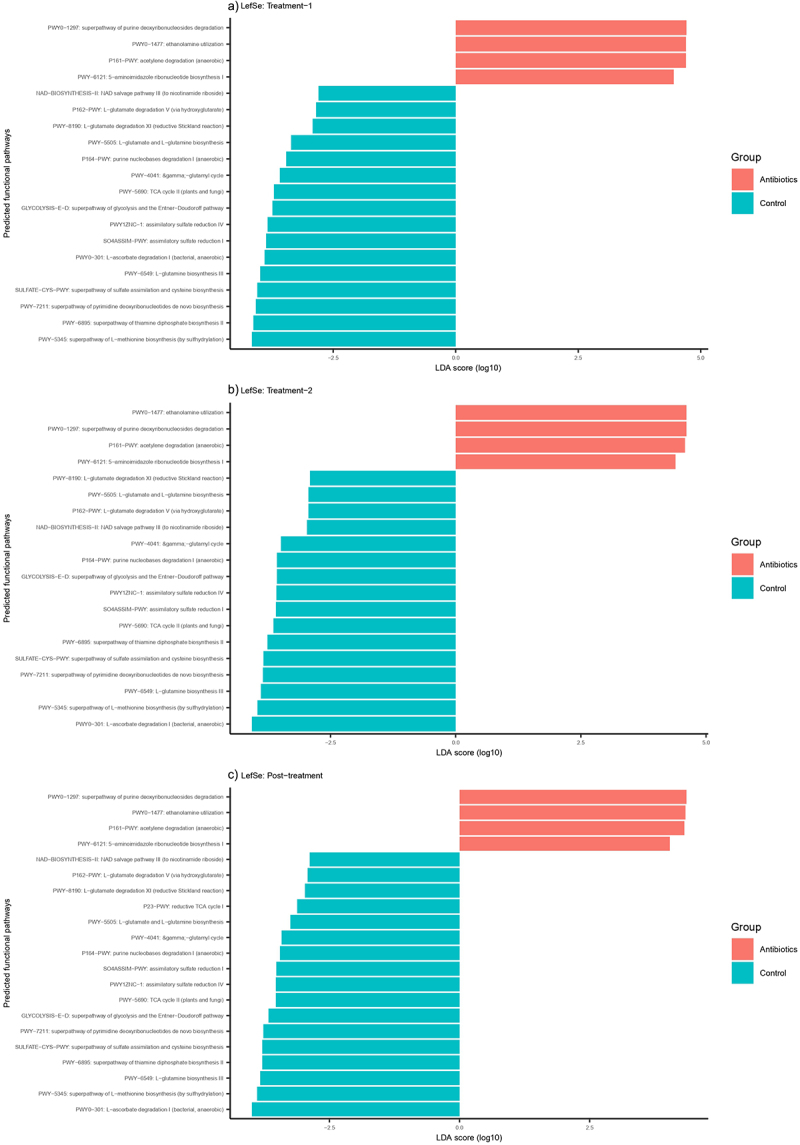
Differentially abundant predicted functional pathways across different phases of the experiment based on LefSe analysis. Only those pathways that were correlated with behaviors (*p* <.05) are displayed here.

Antibiotics altered several metabolic pathways predicted from shotgun metagenomic data. There were 455 functional pathways predicted from shotgun metagenomic data. There were no significant differences between these predicted pathways during pre-treatment phase. One hundred ninety, 148 and 101 of these pathways were altered by antibiotic treatment at treatment-1, treatment-2, and post-treatment phase of the experiment, respectively (Supplementary File 5). To further narrow down the pathways relevant to our study, we first evaluated the correlation between behaviors and the abundances of these pathways. Twenty-nine pathways were significantly correlated with behaviors (*p* < .05) (Supplementary File 5). The top five most significant correlations were all negative correlations between time spent in proximity and PWY-5505: L-glutamate and L-glutamine biosynthesis, PWY-4041: gamma-glutamyl cycle, PWY1ZNC–1: assimilatory sulfate reduction IV, SO4ASSIM-PWY: assimilatory sulfate reduction I, PWY-6895: superpathway of thiamine diphosphate biosynthesis II (*r* = −0.52 to −0.61, *p* < .01). Of these 29 pathways, 20, 20, and 21 pathways were significantly altered at treatment-1, treatment-2, and post-treatment phase, respectively (Figure 5).

### Antibiotics caused changes in the concentrations of fecal metabolites relevant to gut-brain axis communication

**Figure 6.**
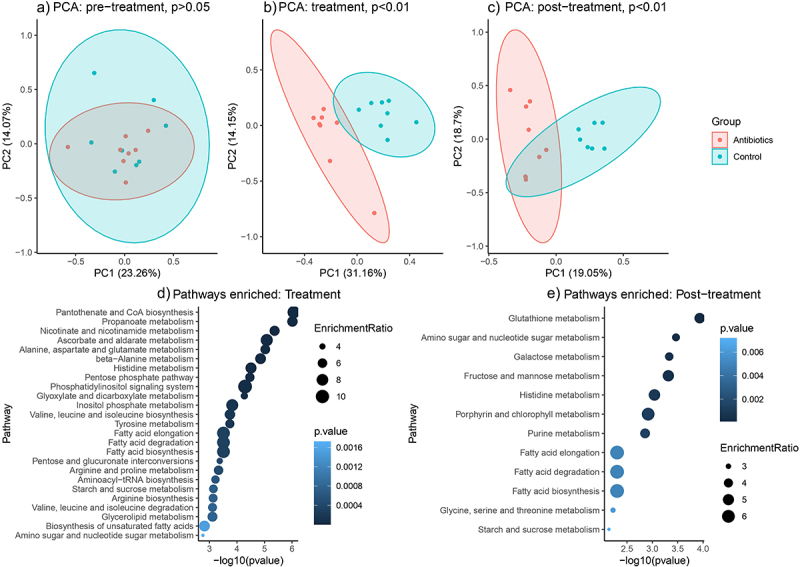
Changes in fecal metabolite and metabolic pathways abundances across time (based on untargeted metabolomics). Figures 5A-C represent principal component analysis plots of the untargeted metabolomic runs for a) pre-treatment phase, b) treatment phase and c) post-treatment phase. Figures 5D-E represent differentially enriched metabolic pathways (top 25) during treatment and post-treatment phases. Metabolic pathways were significantly enriched (p-values after false discovery rate correction < 0.05).

Antibiotics altered the concentrations of numerous metabolites in the gut. There were 196 metabolites detected using an untargeted GC-MS assay. There were no differences between the antibiotic and control groups in the concentrations of these metabolites in the pre-treatment phase. In stark contrast to the pre-treatment phase, there were 103 and 37 metabolites that were differentially abundant between the antibiotic and control groups during treatment and post-treatment phase, respectively (Supplementary File 6). Principal component analysis revealed distinct clusters between the antibiotic and control groups based on relative concentrations of metabolites during treatment and post-treatment phases, but not during the pre-treatment phase (Figures 6a–c).

Pathway enrichment analysis revealed no differences in the abundance of pathways at the pre-treatment phase. There were 53 total pathways mapped to the human KEGG database, of which 46 and 12 pathways were differentially enriched between antibiotic and control groups at treatment and post-treatment phase, respectively (Supplementary File 6). The top 5 differentially enriched pathways during treatment phase were pantothenate and CoA biosynthesis, propanoate metabolism, nicotinate and nicotinamide metabolism, ascorbate and aldarate metabolism, and alanine, aspartate and glutamate metabolism (Figure 6d). The top 5 differentially enriched pathways during post-treatment phase were glutathione metabolism, amino sugar and nucleotide sugar metabolism, galactose metabolism, fructose and mannose metabolism and histidine metabolism (Figure 6e).

**Figure 7.**
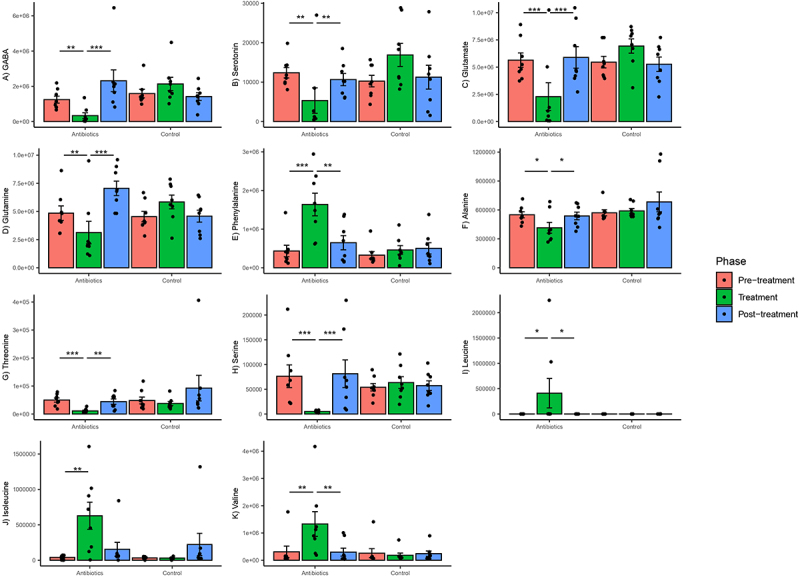
Within-group changes in select fecal metabolites and neurotransmitters (A-K) during the course of the experiment. The y-axes represent normalized abundances of these compounds derived from untargeted GC-MS. Bar-plots represent mean ± standard error. p-values highlighted were estimated from mixed effects general linear models built at the group level. *- p-value <.05, **- p-value <.01, ***- p-value <.001.

Among those metabolites showing a significant time-treatment effect were several key neurotransmitters and amino acids that have been implicated in gut-brain axis communication. Significant group–phase interactions were estimated for GABA, serotonin, glutamate, glutamine, alanine, threonine, serine, leucine, isoleucine, valine, and phenylalanine (Supplementary File 4). There was a significant decrease in relative concentrations of GABA, serotonin and glutamate and certain amino acids (glutamine, alanine, threonine, and serine) in the antibiotics group in the treatment phase relative to pre-treatment phase (Figure 7). There was also a significant increase in relative concentrations of branched chain amino acids (leucine, isoleucine, and valine) and phenylalanine in the antibiotics group in the treatment phase relative to pre-treatment phase (Figure 7), suggesting a significant effect of treatment on branched-chain amino acid biosynthesis. None of these changes persisted into the post-treatment phase, suggesting that the effect was contemporary with treatment and short-lived. There were no changes in the concentrations of these metabolites in the control group (Figure 7).

### Antibiotics caused changes in the concentrations of gut bile acids

**Figure 8.**
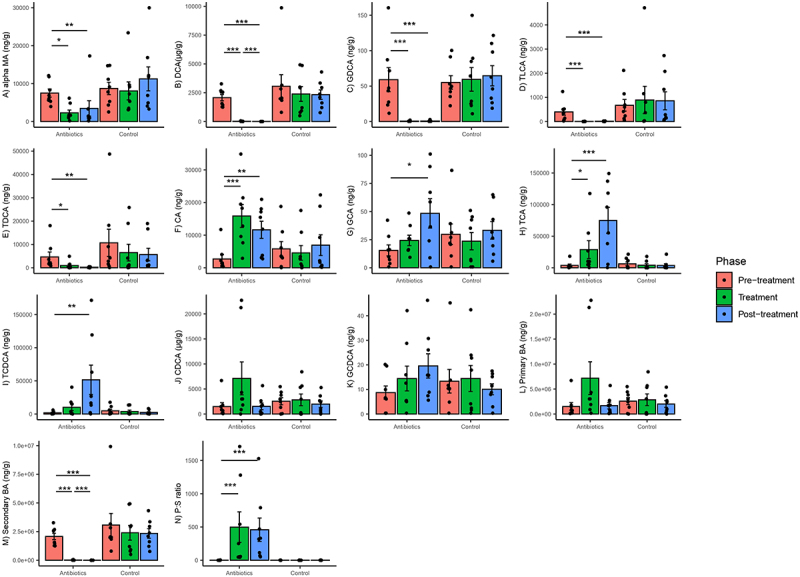
Within-group changes in fecal bile acids concentrations (A-N) during the course of the experiment. Bar-plots represent mean ± standard error. p-values highlighted were estimated from mixed effects general linear models built at the group level. *- p-value <.05, **- p-value <.01, ***- p-value <.001. “BA”- bile acids, “P:S ratio”- Primary:secondary bile acids ratio.

Antibiotics altered the concentrations of several bile acids in the gut. Based on mixed-effects models, there was a significant group–phase interaction estimated for changes in the concentrations of all bile acids and bile acid pools (primary, secondary, primary:secondary bile acid ratios). targeted except for CDCA and GCDCA (Supplementary File 4). There was a significant decrease in αMA, DCA, GDCA, TLCA and TDCA concentrations in the antibiotics group at treatment phase relative to pre-treatment phase and these differences persisted in the post-treatment phase (Figure 8). There was a significant increase in CA and TCA concentrations in the antibiotics group in the treatment phase relative to pre-treatment phase and this increase persisted in the post-treatment phase (Figure 8). Finally, there was a significant increase in GCA and TCDCA concentrations in the antibiotics group in the post-treatment phase relative to pre-treatment phase but not during treatment phase (Figure 8). Bile acid concentrations did not change in the control group (Figure 8). There were no within-group differences in either treatment group in primary bile acids on post-hoc testing. There was a significant decrease and increase in secondary bile acids and primary: secondary bile acid ratios (P:S ratio), respectively, at the treatment phase only in the antibiotic group, and this difference persisted in the post-treatment phase (based on post-hoc tests) (Figure 8).

There was a significant increase in the choloylglycine hyrolase (a bile salt hydrolase) genes in the treatment phase in the antibiotic group, but these levels returned to baseline values in post-treatment phase (based on compound poisson linear model in Maaslin2) (Supplementary File 4). There were no changes in this gene abundance between the different phases of control group (Supplementary File 4). This gene was mapped to *Bacteroides* (*B. ovatus*, *B. caccae*, *B. dorei*, *B. thetaiotamicron*, *B. xylanisolvens*, *B. vulgatus*, *B. intestihominis*), *Coprobacter fastidiosus*, *Parabacteroides merdae* and *Enterococcus* (*E. casseliflavus*, *E. faecium*, *E. gallinarum*, *E. saccharolyticus*) genomes.

### Antibiotics caused changes in the concentrations of SCFAs

**Figure 9.**
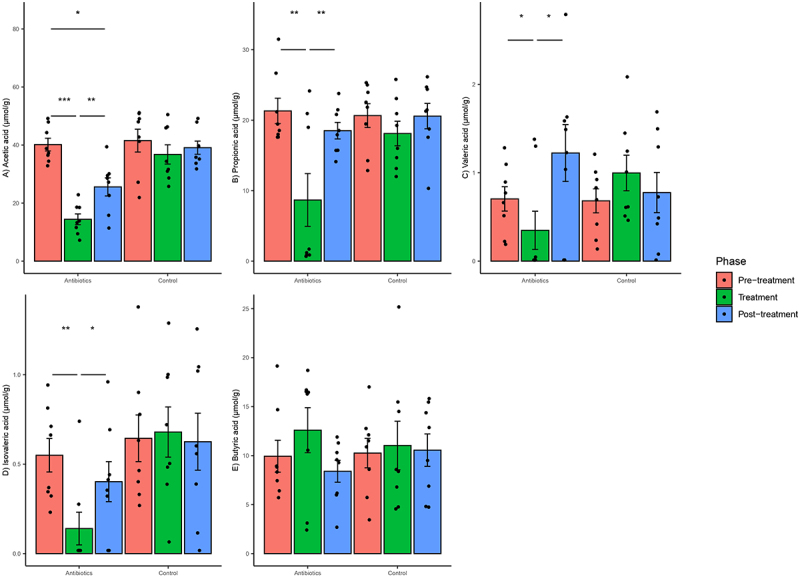
Within-group changes in fecal short-chain fatty acids concentrations (a-e) during the course of the experiment. Bar-plots represent mean ± standard error. p-values highlighted were estimated from mixed effects general linear models built at the group level. *- p-value <.05, **- p-value <.01, ***- p-value <.001.

Antibiotic treatment altered the concentrations of fecal SCFAs. Based on mixed-effects models, there was a significant group–phase interaction estimated for changes in acetic acid, propionic acid, valeric acid and isovaleric acid concentrations but not for butyric acid concentrations (Supplementary File 4). There was a significant decrease in acetic acid concentrations in the antibiotics group in the treatment phase relative to pre-treatment phase and this difference persisted in the post-treatment phase (Figure 9). There was a significant decrease in propionic acid, valeric acid and isovaleric acid concentrations in the antibiotics group as compared to the control group in the treatment phase relative to pre-treatment phase (Figure 9). However, there was no difference in these concentrations in the post-treatment phase, indicating that the altered concentrations of these three SCFAs in the antibiotic group returned to levels seen in the pre-treatment phase. The concentrations of SCFAs did not change in the control group (Figure 9).

### Changes in urinary cortisol at baseline and after exposure to stressor

**Figure 10.**
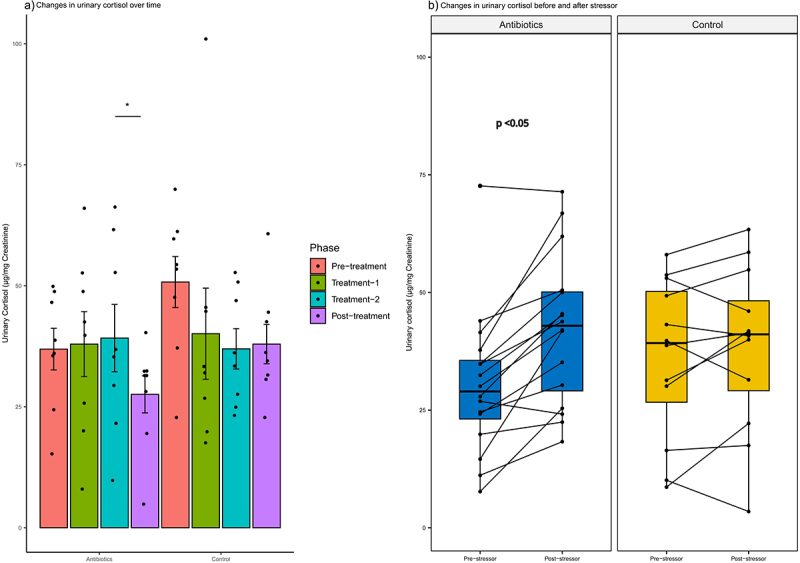
Within-group changes in the urinary cortisol concentrations: a) during the course of experiment and b) before and after exposure to a stressor. Bar-plots represent mean ± standard error. p-values highlighted were estimated from mixed effects general linear models built at the group level. *- p-value <.05, **- p-value <.01, ***- p-value <.001.

Antibiotic treatment had no effect on baseline cortisol concentrations but did produce differential responses in stressor-associated changes in cortisol. Cortisol concentrations in first-void urine samples collected differed between treatment groups throughout the study phases (group-phase interaction, *p* = .046) (Supplementary File 4). Cortisol concentrations tended to be higher in the control group than in the antibiotic group in the pre-treatment phase of the study (*p* = .06). There were no gender differences in the pattern of cortisol levels between groups or across study phases. There was a statistically significant decrease in the urinary cortisol concentrations in the antibiotics group between treatment-2 and post-treatment phase (Figure 10a). There were no differences in the urine cortisol levels across different phases in the control group (Figure 10a).

Exposure to multiple stressors (capture, manual restraint, and isoflurane anesthesia) altered cortisol excretion differently in the antibiotic and control groups (Figure 10b). Relative to non-stress cortisol levels, antibiotic-treated marmosets showed significant elevations (39.4% increase) in cortisol after exposure to stressors (*p* < .05). In contrast, cortisol levels in the control group did not differ as a consequence of exposure to stressors (Figure 10b). As with baseline cortisol concentrations, males and females did not differ in their responses to stressors.

There was a significant negative correlation between urinary cortisol and time spent in proximity (*r* = −0.34, *p* < .05), but there was no significant correlation between urinary cortisol and other behaviors. *Streptococcus* spp. (*r* = −0.38) and unclassified bacteria of family *Prevotellaceae* (*r* = 0.30) were significantly correlated with urinary cortisol at *p* < .05 but these relationships were no longer significant after correcting for multiple comparisons.

### Correlation between gut metabolites, bacterial genera, and behaviors

There were 45 different significant correlations (*p* < .05) between behavior and metabolites, with 16 of these being significant at *p* < .01 (Supplementary File 7). Four behaviors (proximity, interaction, scratching, scent marking) and 41 different metabolites were involved in these correlations, with proximity involved in 35 of these correlations. Both GDCA and DCA were correlated with proximity and interaction. Proximity was additionally correlated with TLCA. None of the SCFAs were part of these correlations. Metabolites other than bile acids and correlated with behavior (*p* < .01) were (in order of strength of correlations): glycerol-2-phosphate, mesoerythritol, glycine, 3-phenylpropionic acid, nonanoic acid (9:0), lactitol, 3-(3-hydroxyphenyl) propionic acid, maleic acid, gluconic acid, lanthionine, benzoic acid, myo-inositol and 2-aminoethanol (Supplementary File 7).

There were 12 significant correlations between bacterial genera (16s rRNA) and behaviors (*p* < .05) (Supplementary File 7). Both proximity and social interaction were correlated with *Oribacterium* spp., unclassified members of family *Muribaculaceae* and order *Bacteroidales*. Proximity was additionally correlated with *Streptococcus* spp., *Fusobacterium* spp., *Olsenella* spp. and unclassified members of families *Enterobacteriaceae* and *Prevotellaceae*. Finally, cage manipulation was correlated with *Collinsella* spp.

**Figure 11.**
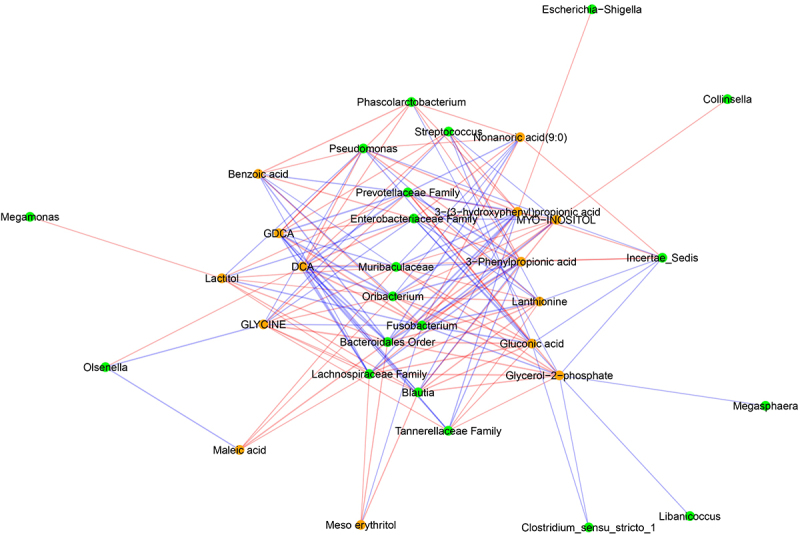
Networks of correlations between fecal metabolites and observed behavior. Red and blue edges represent negative and positive correlations, respectively. Nodes in orange and green represent fecal metabolites and genera based on 16S rRNA sequencing, respectively. Significant correlations (p-values after false discovery rate correction < .01) are presented here.

There were 1443 significant bacterial genera (16s rRNA)-metabolite correlations (*p* < .05) (Supplementary File 7), with 153 of these correlations between bacterial genera and those metabolites that were significantly correlated with behaviors (*p* < .01) (Figure 11). These 153 correlations involved 20 bacterial genera and 14 different metabolites. Top five most influential bacterial genera in terms of correlations with metabolites (*p* < .01) that in turn correlate with behavior were unclassified genera of order *Bacteroidales* (part of 14 correlations), *Fusobacterium* spp., unclassified genera of family *Lachnospiraceae*, family *Prevotellaceae* and family *Muribaculaceae* (part of 13 correlations each).

## Discussion

Several rodent studies have previously demonstrated a link between antibiotic-induced gut dysbiosis and alterations in behavior and response to stress.^13,33,34^ In our study, shotgun metagenomics confirmed the dominance of several key bifidobacterial species characteristic of callitrichids. This is the first study to utilize shotgun metagenomics to obtain species-level resolution in common marmosets and the results here can serve as a baseline for future studies in other colonies. For the first time in a NHP model, this study demonstrated that antibiotics induce gut dysbiosis, alter gut metabolites relevant to gut-brain communication, affect neuroendocrine responses in response to stressful stimuli and change social behavior.

The post-antibiotic changes in the gut microbiome of common marmosets were quite distinct from those observed in rodent models. Reyes et al. (2020) administered a cocktail of antibiotics including neomycin and vancomycin for 14 days to adult mice and reported a decrease in genera of family *Lachnospiraceae* and *Clostridium* spp. without a corresponding increase in abundance of *Fusobacterium* spp.^35^ Hoban et al. (2016) also did not report an increase in Fusobacteria in rats who were administered an antibiotic cocktail (including ciprofloxacin and vancomycin) for 7 weeks.^36^ In our experiments, we specifically observed significant treatment effects on relative abundance of several bacterial taxa (most notable being *Fusobacterium* spp.), which are quite different from those detected in murine models. Differences in the innate gut microbiome composition of common marmosets versus mice might have contributed to the disparate dysbiosis induced by these antibiotic cocktails.

Antibiotics induced a unique gut dysbiosis characterized by an increase in the relative abundance of *Fusobacterium spp*. Fusobacteria are gram-negative, obligate anaerobic bacteria which are present as commensals in oral and gut microbiomes of animals.^37^ The role of Fusobacteria in gastrointestinal disease pathology has garnered more attention recently, with a positive association found between presence of *Fusobacterium nucleatum* and inflammatory and neoplastic diseases of the gastrointestinal tract in humans in several studies.^38^ Relative abundances of *Fusobacterium* spp. have been found to be increased in humans with anxiety disorder.^39–41^ However, we were not able to find literature regarding the gut health or behavioral implications, and bacterial physiology or genomics of *Fusobacterium mortiferum* in common marmosets. The structure and function of this bacteria isolated from common marmosets should be characterized in the future.

There was a significant decrease in the bifidobacterial populations in the gut after antibiotic administration. Higher relative abundances of *Bifidobacterium* spp. is a characteristic of callitrichid gut microbiomes and play a pivotal role in the health of these monkeys by metabolizing key sugar substrates.^42^ *Bifidobacterium* spp. have also been associated with modulating stress responses in animals and humans. Several strains of *Bifidobacterium* spp. have been proven to decrease anxiety and stress and improve neurocognition in both healthy and moderately stressed humans.^43,44^ We postulate that decrease in *Bifidobacterium* spp. might have contributed to the changes in behaviors in these monkeys after antibiotic intervention. There are strong signals that other less prevalent bacterial genera and species were also influential in modulating behavior in marmosets. However, a lack of definitive characterization of these taxons at the species and genera levels due to limitations of existing bacterial genomes and 16s rRNA databases limits further discussion on these microbiome-metabolic-behavior interrelationships.

Urinary cortisol is a reliable indicator of psychological stress in common marmosets.^22^ The changes in urinary cortisol levels should be interpreted in the context of the key behavioral changes observed, especially those observed in the antibiotic-treated group only. There was an increase in the time spent closer to the pair-mate (proximity) and interacting with the pair-mate (social interaction), both examples of affiliative behavior in common marmosets. Increase in cage manipulations can further be interpreted as attempts to establish contact with marmosets in neighboring enclosures.^45^ This behavior is consistent with the theory of “tend-and-befriend” response to stressors and the social models of support proposed by Taylor et al. (2000) and Cohen and Wills (1985), respectively.^46,47^ According to Taylor et al. (2000), certain species of animals will respond to the presence of stressors by demonstrating affiliative behaviors rather than the more widely recognized “fight-or-flight” response.^46^ Cohen and Wills (1985) proposed that affiliative behavior shown by a social partner such as physical contact might ameliorate the harmful consequences of a stressor.^47^ These theories have been backed by the results of several studies conducted to evaluate the response of several stressors in common marmosets.^45,48^ Marmosets placed in a novel enclosure along with the pair-mate exhibited significantly lesser hypothalamus-pituitary-adrenal axis activation as compared to those marmosets which were placed without the pair-mate.^49^ In another study, juvenile marmosets who engaged in social play for longer periods displayed lesser baseline cortisol levels and cortisol reactivity.^50^ It is possible that the physiological and behavioral responses to alterations in the levels of gut-brain axis relevant metabolites might have been buffered by the presence of a well-established social bond with the pair-mate as an adaptive, coping mechanism. Statistical correlation between urinary cortisol and time spent in close proximity in our study provides evidence in favor of this hypothesis. Hence, we postulate that an increase in sociability (proximity and interaction) as an indicator of potential stress induced by gut dysbiosis in the context of this specific study.

These changes in social behavior are unique to this marmoset model because we tested these changes in well-established pair-mates of opposing genders (a characteristic unique to marmosets), whereas the social behavior tested in rodents focus on social preference (preferring another rodent over an object) and social novelty (preferring a novel rodent over a known rodent).^11,51^ Moreover, these experiments are usually performed with only male rodents, which tend to exhibit either an aggressive, fight-or-flight response or avoidance response as compared to the more affiliative, tend-and-befriend response shown by females.^28,52^ The existing literature on rodent models suggest either a decrease in sociability or no change in social behavior post-antibiotic-induced dysbiosis,^11^ whereas our study uniquely presents a case for an increase in sociability after oral antibiotic intervention, albeit as a consequence of potential stress. There were potential changes in piloerection and genital display in control group, but the results of these behaviors were not statistically robust and prone to outlier influence. In the case of genital display, the counts in the control group were largely inflated due to aberrantly high display of this behavior of a single subject. Although the counts of piloerection and genital display were higher in control marmosets, these values remained similar to non-stressed marmosets in other studies^53,54^ and were far lower than those reported for stressed marmosets.^55–57^

Increase in cortisol reactivity after a stressor provides strong evidence that the antibiotic-induced gut perturbations were able to alter the functioning of the HPA axis in a multiple stressor model (strong gut dysbiosis plus excessive handling). The marked increase in cortisol reactivity after multiple stressors is in line with previous studies conducted on marmosets described above^22,48^ and the existing theories that psychiatric disorders in humans result from a combination of different etiological risk factors.^58^ It could also be postulated that strong social-bonding can buffer the stress due to a single stressor, but fails to moderate such stress in the presence of multiple stressors.

The antibiotic treatments in our study also altered the levels of several other gut metabolites such as bile acids and SCFAs that can play a role in gut-brain axis communication. SCFAs are produced by fermentation of complex carbohydrates, such as dietary fiber in the gut.^59^ Decrease in fecal concentrations of SCFAs has been found in patients suffering from autism spectrum disorder, Alzheimer’s disease and chronic stress.^60^ Mechanisms by which SCFAs play a role in gut-brain axis communication have been elucidated in rodent models, where SCFAs seem to facilitate communication by modulating signaling of vagus nerve, binding to the G-protein coupled receptors in the gut and the brain and modulating intracellular responses, binding to the receptors of enteroendocrine cells to modulate secretions of neuropeptides and decreasing neuroinflammation and gut inflammation.^60^ However, the impact of SCFAs on behavior was more limited in contrast to the impact of bile acids, certain amino acids, and other metabolites.

We also found elevation in certain fecal primary bile acids (CA, GCA, TCA and TCDCA) and decrease in fecal secondary bile acids (DCA, GDCA, TLCA, and TDCA). These combined data suggest a failure of intestinal bacteria to deconjugate primary bile acids and synthesize secondary bile acids, leading to the reabsorption of primary bile acids into the enterohepatic circulation.^61^ Additionally, primary bile acid biosynthesis pathway was differentially enriched in the antibiotic group during the treatment phase. This increase in systemic reserves of untransformed bile acids might also explain the longer persistence in bile acid alterations as compared to the more transient nature of changes in other metabolites. Interestingly, we also observed an increase in a gene encoding bile salt hydrolase (choloylglycine hydrolase) in the gut microbiome of antibiotic treated subjects. Bile salt hydrolases mediate microbial deconjugation of bile acids released into the intestines from liver (i.e., conversion of GCA, TCA, TCDCA and GCDCA back to CA and CDCA).^62^ The bacterial species associated with these genes were not differentially abundant between treatment groups, with the exception of *Parabacteroides merdae, Bacteroides ovatus* and *Bacteroides xylanisolvens*. Increased conjugated primary bile acids might have exerted a selective pressure on gut bacteria capable of “detoxifying” these compounds. However, this relationship could not be formally analyzed because of a large number of genes unmapped to any bacterial species. Deconjugation of bile acids leads to the release of glycine and taurine, which are used as energy source by some bacteria.^63,64^ Subsequent release of unconjugated bile acids inhibit certain bacteria by causing cell membrane damage and play a role in maintaining gut microbial homeostasis.^63,64^ Hence, altered bile acid levels might have contributed to gut dysbiosis.

The increase in fecal primary:secondary bile acid ratio observed in our study is also a characteristic of irritable bowel syndrome (IBS) and Crohn’s disease in humans.^62,65^ Wei et al. (2020) reported decreased alpha diversity, higher fecal P:S ratio, higher fecal CA, TCA, GCA, TCDCA, and GCDCA, and higher anxiety and depression score in patients with IBS as compared to healthy people.^62^ Feng et al. (2022) reported nearly identical results in Crohn’s patients with the addition of lower DCA in these patients and found statistical correlations between bile acids and psychological disorders.^65^ Recent studies in rodents have provided more mechanistic insights into the relationship between fecal bile acids and psychological disorders. Golubeva et al. (2017) compared differences in several biological parameters between C57BL/6 and BTBR (a model for autism) mice, and reported an increase in conjugated bile acids and anxiety in BTBR mice.^66^ Intestinal dysmobility and impaired gut barrier function were postulated to be caused by altered bile acid concentrations in intestine.^66^ Secondary bile acids also play a direct role in modulating gut inflammation by activation of Farnesoid X receptors (FXR) and G-protein coupled bile acid receptors.^65^ Intestinal dysmobility, gut leakiness and pro-inflammation have all been established to play a role in gut-brain-axis communication.^18^ In our study, levels of secondary bile acids (GDCA and DCA) were correlated with time spent in proximity. These bile acids have also shown to have direct influence on central nervous system by passively diffusing across blood–brain barrier and binding to FXR and exerting an anti-depressant effect.^61^ Knowledge regarding the impact of bile acids on behavior and psychological disorders is constantly evolving and more studies are needed to conclusively define gut microbiome-bile acid–brain relationship.

Similarly, we also detected treatment-associated effects on gut microbiome associated neurotransmitters and amino acids that are precursors of neurotransmitters. Antibiotics decreased fecal levels of serotonin, GABA, glutamate, and glutamine in our study. Serotonin is a mood-modulating, monoamine neurotransmitter, and can be produced both centrally and peripherally.^67^ The importance of gut serotonin levels on the functions of the brain has been questioned since the peripheral serotonin cannot cross the blood–brain barrier.^68^ However, recent studies using germ-free mice have shown that fecal serotonin levels might correlate with central serotonin levels and anxiety-like behaviors.^69^ Fecal serotonin concentrations are also correlated negatively with perceived stress levels in children.^70^ GABA and glutamine are the major inhibitory and excitatory neurotransmitters in the mammalian cortex, respectively.^71^ Levels of GABA and glutamate are intrinsically linked as GABA is synthesized from glutamate.^71^ Alterations in putative pathways associated with the synthesis or conversion of GABA, glutamate, and glutamine inferred from shotgun metagenomics (PWY-6549, PWY-8190, P162-PWY, PWY-5505, PWY-4041, PWY-5690) confirm that concentrations of some of these neurotransmitters or their precursors were modulated by gut bacteria. Metabolic pathways found to be differentially enriched using untargeted fecal metabolomics (propanoate, histidine, beta-alanine, arginine, and proline metabolism) have also been associated with depression in human patients.^72^ Some of these pathways also involve glutamate as an intermediary product. These pathways were also correlated with some behaviors, completing the trifecta of gut microbiome-metabolome-behavior relationship. A recent study has reported decreased levels of fecal-derived GABA and altered phenylalanine metabolism in patients suffering from major depressive disorder.^73^ A meta-analysis of eight cohorts found an association between elevated glutamine degradation and decreased glutamate and leucine synthesis in the gut with depression in humans.^74^ Branched chain amino acids (isoleucine, leucine and valine) were elevated in the gastrointestinal tracts of antibiotic-treated marmosets. These amino acids are primary substrates for gut bacteria for the production of SCFAs such as valeric and isovaleric acid.^75^ High concentrations of these amino acids further indicate that normal gut bacterial physiology was disturbed leading to either decreased uptake or utilization of these amino acids. These findings further lend credence that this model of antibiotic-induced dysbiosis can be used to study gut-brain axis communication in common marmosets in future studies.

Some of the compounds detected by untargeted metabolomics were also significantly correlated with behaviors, particularly with time spent in proximity. These can serve as novel diagnostic biomarkers or treatments of psychiatric diseases. Lanthionine, a potentially uremic toxin and a by-product of cysteine metabolism, has been shown to alter normal behavior in Zebrafish model.^76^ On the other hand, lactitol is a non-absorbable disaccharide which has been used an adjunct therapeutic for hepatic encephalopathy and can modulate gut microbiome.^77^ Benzoic acid has been proposed as an antidepressant because of its capability to cross the blood–brain barrier and exert antidepressant effect by directly inhibiting D-amino acid oxidase.^78^

In summary, we were able to show that antibiotic-induced gut dysbiosis can lead to behavioral, metabolic, and endocrine changes relevant to gut-brain axis communication. This study should serve as a first step in developing a pre-clinical, NHP model for studying the effect of antibiotics on gut-brain axis communication. Implementing more complex behavioral or social paradigms such as exposure to novel objects or dummy predators and social isolation in addition to feeding antibiotics can further generate more valuable data on how gut dysbiosis affects cognition and response to stress and fear in marmosets. Ancillary testing such as cytokine and metabolomic profiling of serum or cerebrospinal fluid and more invasive testing such as histology and transcriptomics of sections of brain tissue are needed to conclusively determine how gut dysbiosis leads to changes in the gut-brain communication in marmosets.

## Supplementary Material

hayer_et_al_gut_microbes_supplementary_material_4_revision.docx

hayer_et_al_gut_microbes_supplementary_material_1_revision.docx

hayer_et_al_gut_microbes_supplementary_material_3_revision.docx

hayer_et_al_gut_microbes_supplementary_material_5_revision.xlsx

hayer_et_al_gut_microbes_supplementary_material_8.docx

hayer_et_al_gut_microbes_supplementary_material_6_revision.xlsx

hayer_et_al_gut_microbes_supplementary_material_7_revision.xlsx

hayer_et_al_gut_microbes_supplementary_material_2_revision.docx

## Data Availability

The 16S rRNA gene amplicon sequence data reported in this paper have been deposited in the European Nucleotide Archive (ENA) at EMBL-EBI under accession number PRJEB61190.
