# Supplementary material for: Antibiotic-induced gut dysbiosis elicits gut-brain axis relevant multi-omic signatures and behavioral and neuroendocrine changes in a nonhuman primate model
Source: Gut Microbes. 2024 Jan 29;16(1):2305476. doi: 10.1080/19490976.2024.2305476 (PMC10826635; doi:10.1080/19490976.2024.2305476)
Supplement: hayer_et_al_gut_microbes_supplementary_material_4_revision.docx [file KGMI_A_2305476_SM1822.docx]

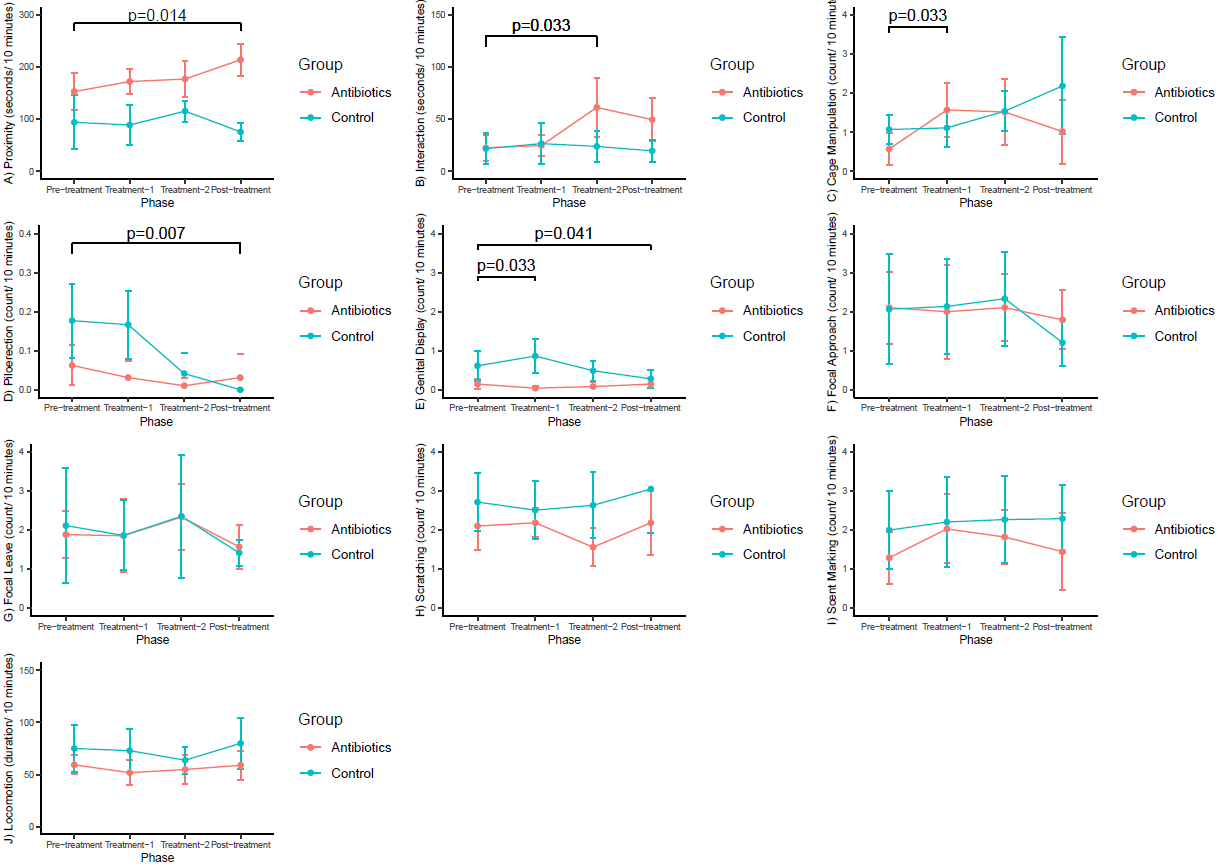
Changes in behavior during the course of the experiment (line-plots). p-values are highlighted whenever there is a significant group-time interaction (p <0.05) with relation to pre-treatment phase and control group (reference points) in mixed-effects models. Error bars represent 95% confidence intervals.


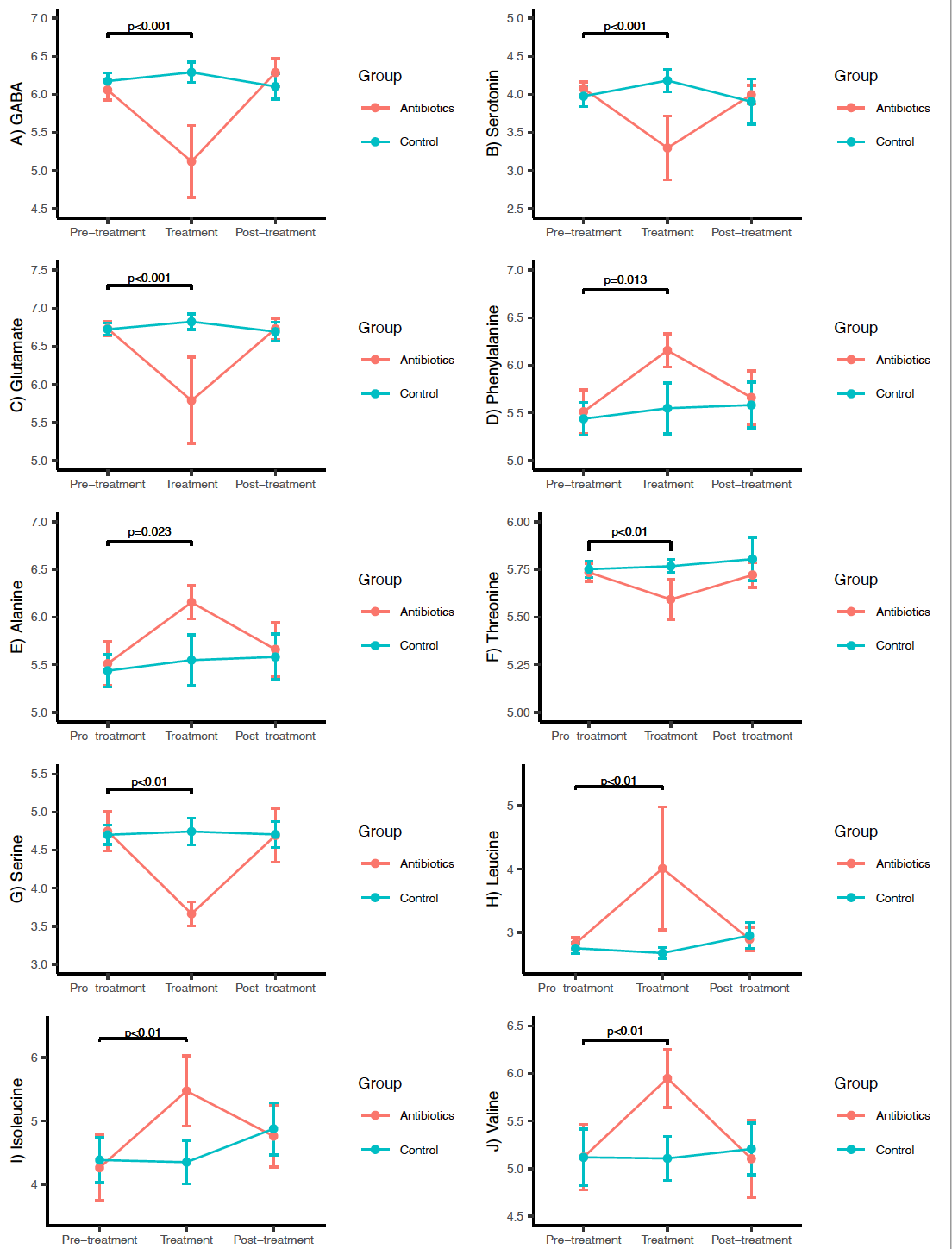


Changes in select gut metabolites and neurotransmitters during the course of the experiment (line-plots). Y- axes represent log10 transformed normalized abundances obtained from untargeted GC-MS spectrometry. p-values are highlighted whenever there is a significant group-time interaction (p <0.05) with relation to pre-treatment phase and control group (reference points) in mixed-effects models. Error bars represent 95% confidence intervals.


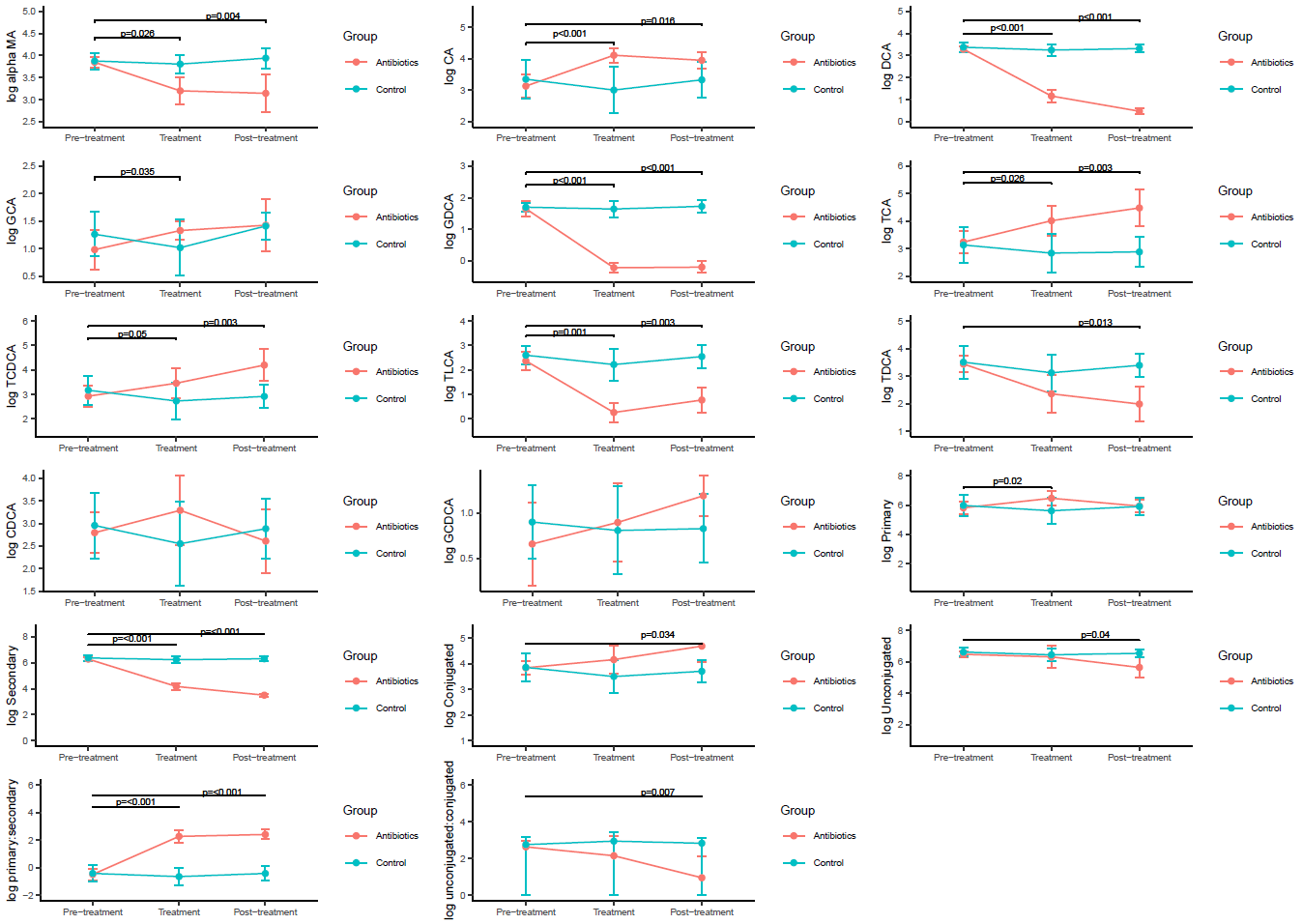


Changes in gut bile acids during the course of the experiment (line-plots). Y-axes represent log10 transformed concentrations. p-values are highlighted whenever there is a significant group-time interaction (p <0.05) with relation to pre-treatment phase and control group (reference points) in mixed-effects models. Error bars represent 95% confidence intervals.


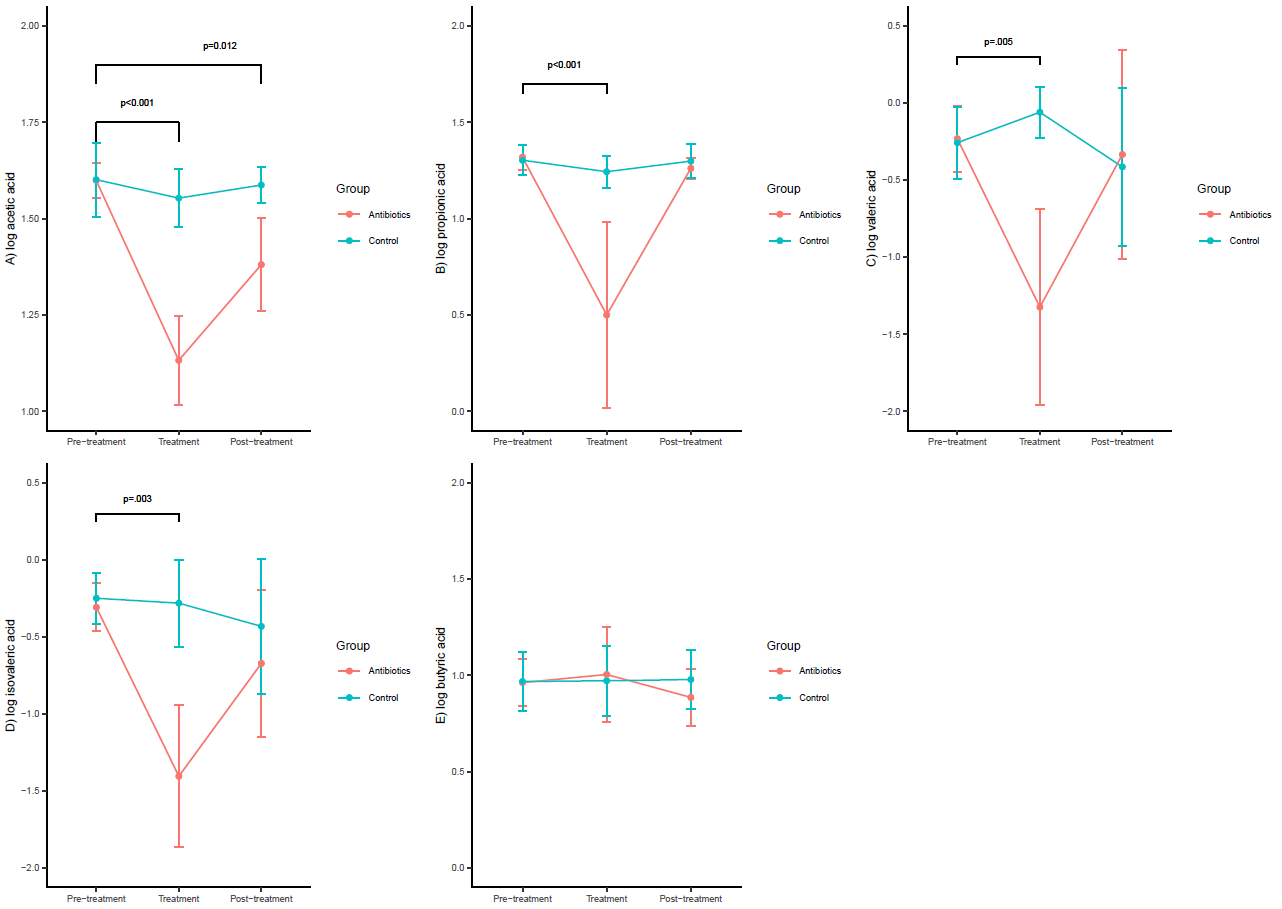


Changes in gut short chain fatty acids during the course of the experiment (box-plots). Y-axes represent log10 transformed concentrations. p-values are highlighted whenever there is a significant group-time interaction (p <0.05) with relation to pre-treatment phase and control group (reference points) in mixed-effects models. Error bars represent 95% confidence intervals.


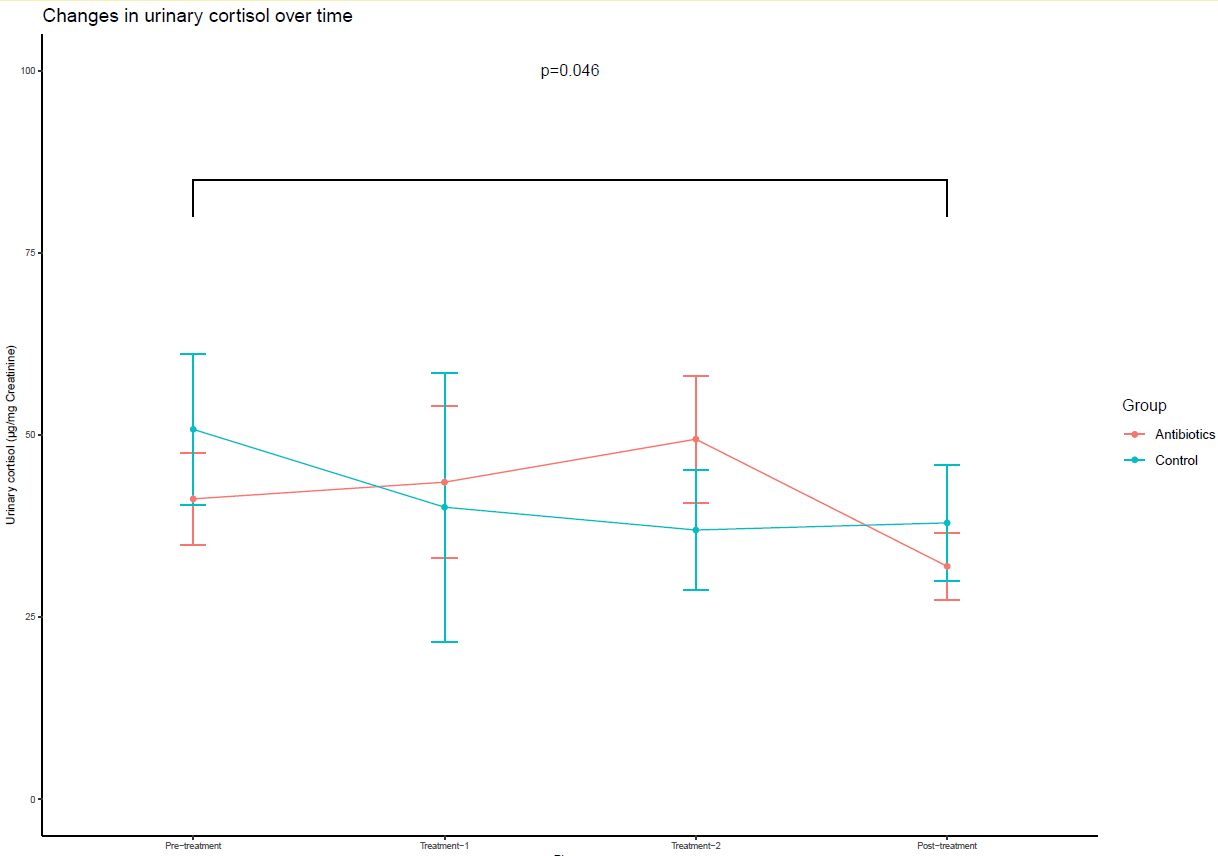


Changes in urinary cortisol during the course of the experiment (box-plots). p-values are highlighted whenever there is a significant group-time interaction (p <0.05) with relation to pre-treatment phase and control group (reference points) in mixed-effects models. Error bars represent 95% confidence intervals.


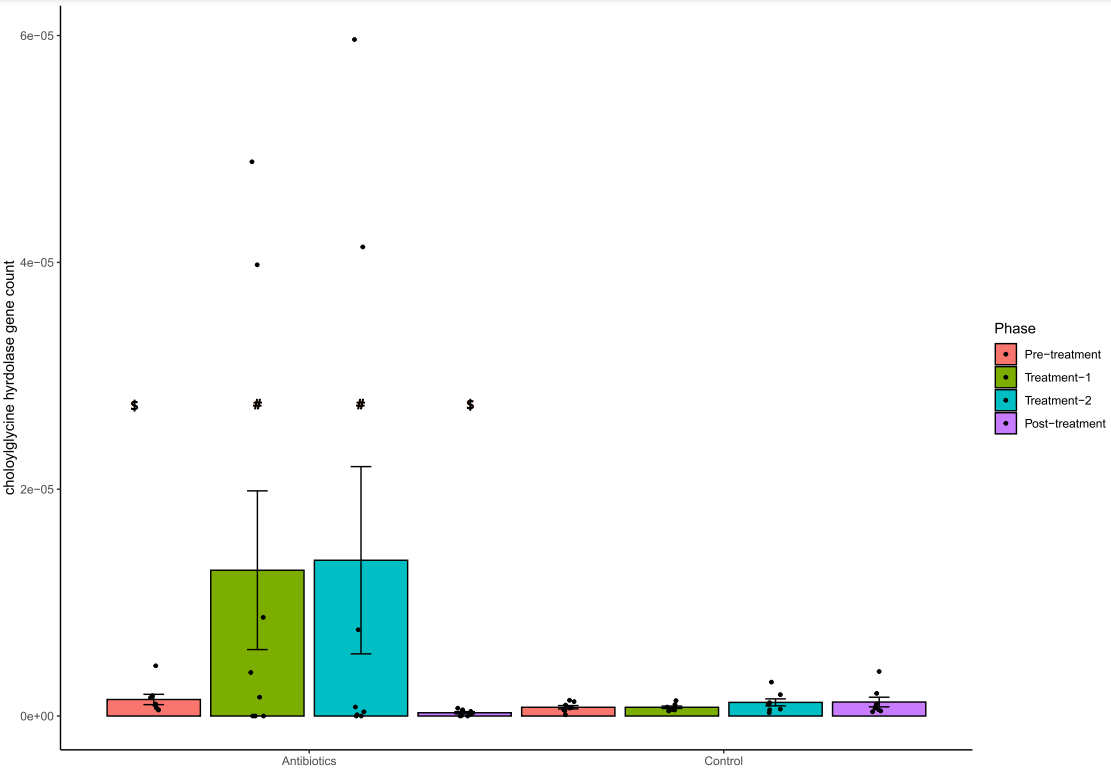


Choloylglycine hydrolase gene content was significantly higher in Treatment-1 and Treatment-2 phases of the antibiotic group as compared to pre-treatment (Maaslin2, CPLM model). There were no significant changes in gene content in control group.
