# Supplementary material for: Antibiotic-induced gut dysbiosis elicits gut-brain axis relevant multi-omic signatures and behavioral and neuroendocrine changes in a nonhuman primate model
Source: Gut Microbes. 2024 Jan 29;16(1):2305476. doi: 10.1080/19490976.2024.2305476 (PMC10826635; doi:10.1080/19490976.2024.2305476)
Supplement: hayer_et_al_gut_microbes_supplementary_material_1_revision.docx [file KGMI_A_2305476_SM1821.docx]

**Untargeted metabolomics using GC-MS:** An aliquot of 50 mg of fecal sample was extracted using a chilled solution of water:chloroform:methanol (3:5:12) spiked with 16 µg of pinitol as the internal standard. The samples were disrupted and homogenized by adding 2 stainless steel beads (SSB 32) using the TissueLyserII (Qiagen, Germantown, MD, USA) at 25 Hz for 3 min. After centrifugation at 16,000 g for 10 min at 4°C, the supernatants were collected and transferred to a new tube. The samples were back extracted using the same solution and the supernatant collected after centrifugation was combined to the first one. To separate the water/methanol phase from the chloroform, a chilled solution of water:chloroform (3:4.5) was added to the supernatant, vortexed and centrifuged for 10 min at 16,000 g at 4°C. The upper phase was transferred to a new tube and dried down using a SAVANT speed-vac. A small aliquot of each sample was pooled together to make a quality control (QC) sample. The samples were then resuspended in 20 mg/mL methoxyamine hydrochloride reagent prepared in pure pyridine and incubated for 2 hr at 37°C on a platform shaker at 1000 rpm. Next, for derivatization, MSTFA +1% TMCS (ThermoFisher Scientific) was added to each sample, incubated for 30 min at 37°C on a platform shaker at 1000 rpm followed by a centrifugation for 10 min at 16,000 g prior to transferring the mixture to GC vials. The GC-MS analysis was carried out using an Agilent GC (Model 7890B) and MS Quadrupole (Model 5977A) (Agilent Technologies, Santa Clara, CA, USA). The liquid injection was done using a PAL System RSI 85 (PAL, Lake Elmo, MN, USA). The injector temperature was 260°C; the MS transfer line was 230°C. Metabolites were separated on a HP-5MS column (30 m x 0.25 mm, 0.25 μm, Agilent Technologies), at constant flow of 1.5 ml.min-1 of helium as a carrier gas. One microliter of the derivatized sample was injected into the injector operating in 1:4 split mode. The temperature of the column was initially set to 60°C, and increased at a rate of 10°C.min−1 to 325°C, followed by a hold for 10 min. The samples were run alongside the mixture of retention index C10-25 alkanes (DRO Mix Tennessee/Mississippi) for library search. The identification and quantification of the data was performed using MS-DIAL v4.48, which includes the deconvolution and alignment steps of the peaks. Two libraries were used, a local library made from running authentic standards with Kovats RI, and a public spectrum library, the curated Kovats RI with a total of 28,220 compounds (which includes the Fiehn, RIKEN and MoNA databases). The peaks were reviewed, and a final list of compounds with RI similarities >95% were reported. The peak area was normalized based on the internal standard spiked in the samples during extraction and using LOWESS (locally weighted scatterplot smoothing) for QC-batch normalization. (DRO Mix Tennessee/Mississippi) for library search. The identification and quantification of the data was performed using MS-DIAL v4.48, which includes the deconvolution and alignment steps of the peaks. Two libraries were used, a local library made from running authentic standards with Kovats RI, and a public spectrum library, the curated Kovats RI with a total of 28,220 compounds (which includes the Fiehn, RIKEN and MoNA databases). The peaks were reviewed, and a final list of compounds with RI similarities >95% were reported. The peak area was normalized based on the internal standard spiked in the samples during extraction and using LOWESS (locally weighted scatterplot smoothing) for QC-batch normalization.

**Short chain fatty acids (SCFAs) assay using GC-MS:** An aliquot of 50 mg of fecal sample was extracted using 0.5% phosphoric acid spiked with 83.7 µg of D3-acetate as the internal standard. The samples were disrupted and homogenized by adding 2 stainless steel beads (SSB 32) using the TissueLyserII at 20 Hz for 2 min. The samples were additionally sonicated for 5 min. After centrifugation at 16,000 g for 10 min, the supernatants were transferred to a new tube. Butanol was added to the supernatant, and samples were extracted one more time using the TissueLyserII at 2 Hz for 2 min. The samples were centrifuged at 16,000 g for 10 min and the upper phase was transferred to a new tube. A small aliquot of each sample was pooled together to make a quality control (QC) sample. The samples were transferred to GC vials and injected without derivatization. The GC-MS analysis was carried out using the same equipment described in the untargeted metabolomics section. The injector temperature was 250°C; the MS transfer line was 230°C. Metabolites were separated on a VF-WAXms column (30 m x 0.25 mm, 0.25 μm, Agilent Technologies), at constant flow of 1.2 ml.min-1 of helium as a carrier gas. One microliter of sample was injected into the injector operating in 1:2 split mode. The temperature of the column was initially set to 70°C, and increased at a rate of 12°C.min−1 to 170°C, and then at 25°C.min−1 to 250°C, followed by a hold for 10 min. The acquisition was set up as a SIM (Single Ion Monitoring) scan method using selected ions to analyze the detectable SCFAs (D3-acetate, 46-63 ions; acetate, 43-60 ions; propionate, 45-74 ions; butyric acid, 60-73 ions; isovaleric acid, 60-74 ions; valeric acid, 60-73 ions). The data was acquired at a scan speed of 3.125 u/s with a dwell time of 30 ms for each ion selected. The generated data was analyzed with Agilent MassHunter Quantitative Analysis. For quantification, an external standard curve was prepared using a series of standard samples containing different concentrations of SCFAs and fixed concentration of the internal standard.

**Bile acids assay using LC-MS/MS:** An aliquot of 50 mg of fecal samples was extracted by adding 2 stainless steel beads (SSB 32) and chilled methanol:acetonitrile (1:1) solution using the TissueLyserII at 20 Hz for 3 min. The internal standard used is a mixture of several isotope labelled bile acids (D4-taurochenodeoxycholic acid; D4-taurocholic acid; D4-glycocholic acid; D4-glycochenodeoxycholic acid; D4-chenodeoxycholic acid; D4-deoxycholic acid). Samples were centrifuged at 4°C at 16,000 g for 10 min, and supernatants were transferred to new tubes. Samples were extracted the same way a second time with supernatants combined to the first one and then dried down using a SAVANT speed-vac. Pellets were resuspended using 30% methanol and transferred to HPLC vials. The samples were analyzed by LC-MS/MS using Multiple Reaction Monitoring (MRM) scan mode. The UPLC Nexera X2 (Shimadzu, Columbia, MD, USA) system used was interfaced with a QTRAP 6500+ (Sciex, Redwood City, CA, USA) mass spectrometer equipped with a TurboIonSpray (TIS) electrospray ion source. Analyst software (version 1.6.3) was used to control sample injection, separation, acquisition and data analysis. Bile acids were separated using the ACCQ-TAG ULTRA C18 (2.1 x 100 mm, 1.7 µm, Waters) running at a flow rate of 0.4 mL/min. The gradient of the mobile phases A (2 mM ammonium formate/0.5% formic acid) and B (0.5% formic acid/10% isopropanol/90% acetonitrile) was as follow: 30% B for 1.5 min, to 55% B in 0.2 min, to 98% B in 3.3 min, hold at 98% B for 5 min, to 30% B in 0.5 min. The column compartment was set at 55°C. The QTRAP 6500+ mass spectrometer was tuned and calibrated according to the manufacturer’s recommendations. The mass spectrometer was operated with the IonDrive Turbo V electrospray ionization (ESI) source in negative ion mode. The ESI source operation parameters were as follows: source temperature at 400°C; ion spray voltage at –4500; ion source gas 1 at 40; ion source gas 2 at 40; curtain gas at 20 psi; collision gas at medium. The bile acids were detected using MRM transitions that were optimized using standards. The MRM transition (Q1-Q3) for the compounds are as follow: α-muricholic acid (407-387), β-muricholic acid (407-371), chenodeoxycholic acid (391-373), cholic acid (407-343), deoxycholic acid (391-345), glycochenodeoxylic acid (448-74), glycocholic acid (464-74), glycodeoxycholic acid (448-74), glycolithocholic acid (432-74), hyocholic acid (407-389), lithocholic acid (375-357), ω-muricholic acid (407-387), taurochenodeoxycholic acid (498-80), taurocholic acid (514-80), taurodeoxycholic acid (498-80), taurolithocholic acid (482-80). For quantification, an external standard curve was prepared using a series of standard samples containing different concentrations of unlabeled compounds and fixed concentrations of the internal standards.
