# Supplementary material for: Antibiotic-induced gut dysbiosis elicits gut-brain axis relevant multi-omic signatures and behavioral and neuroendocrine changes in a nonhuman primate model
Source: Gut Microbes. 2024 Jan 29;16(1):2305476. doi: 10.1080/19490976.2024.2305476 (PMC10826635; doi:10.1080/19490976.2024.2305476)
Supplement: hayer_et_al_gut_microbes_supplementary_material_3_revision.docx [file KGMI_A_2305476_SM1820.docx]

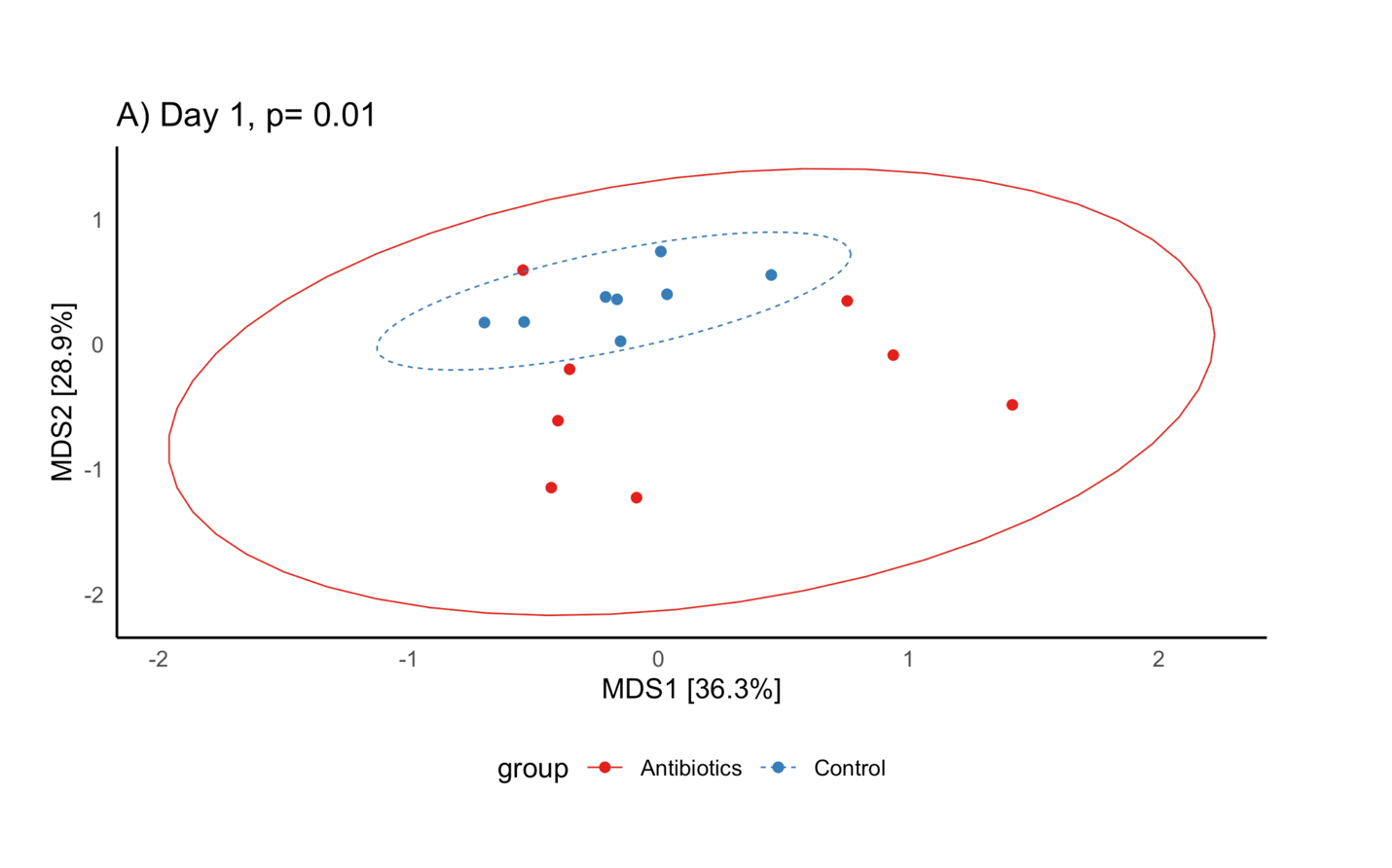


A) Beta diversity analyses, specifically PERMANOVA analysis of Bray-Curtis distances, revealed significant differences in population structure of bacterial genera on the first day of the pre-treatment phase (day 1).


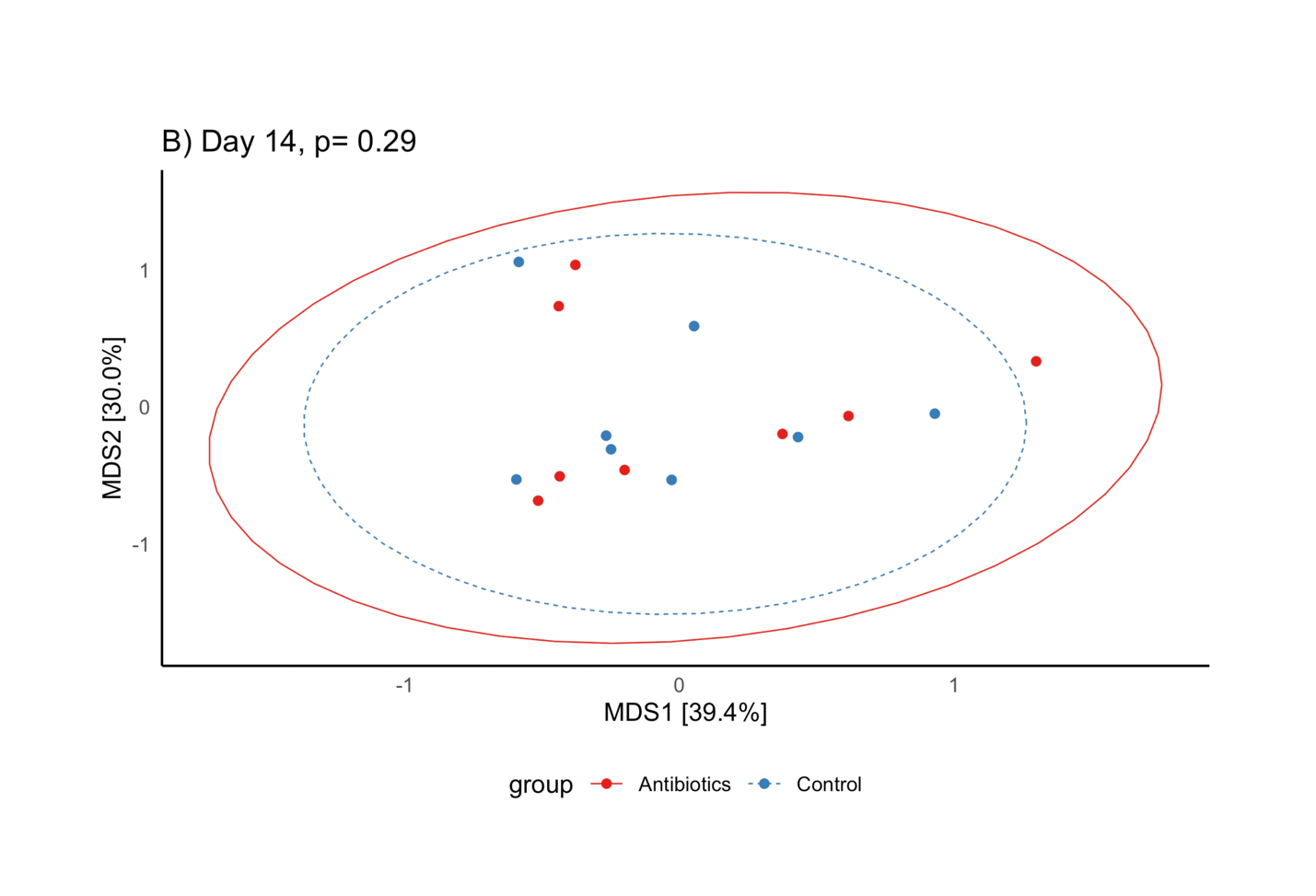


B) Beta diversity analyses, specifically PERMANOVA analysis of Bray-Curtis distances, did not reveal significant differences in population structure of bacterial genera on the last day of the pre-treatment phase (day 14).


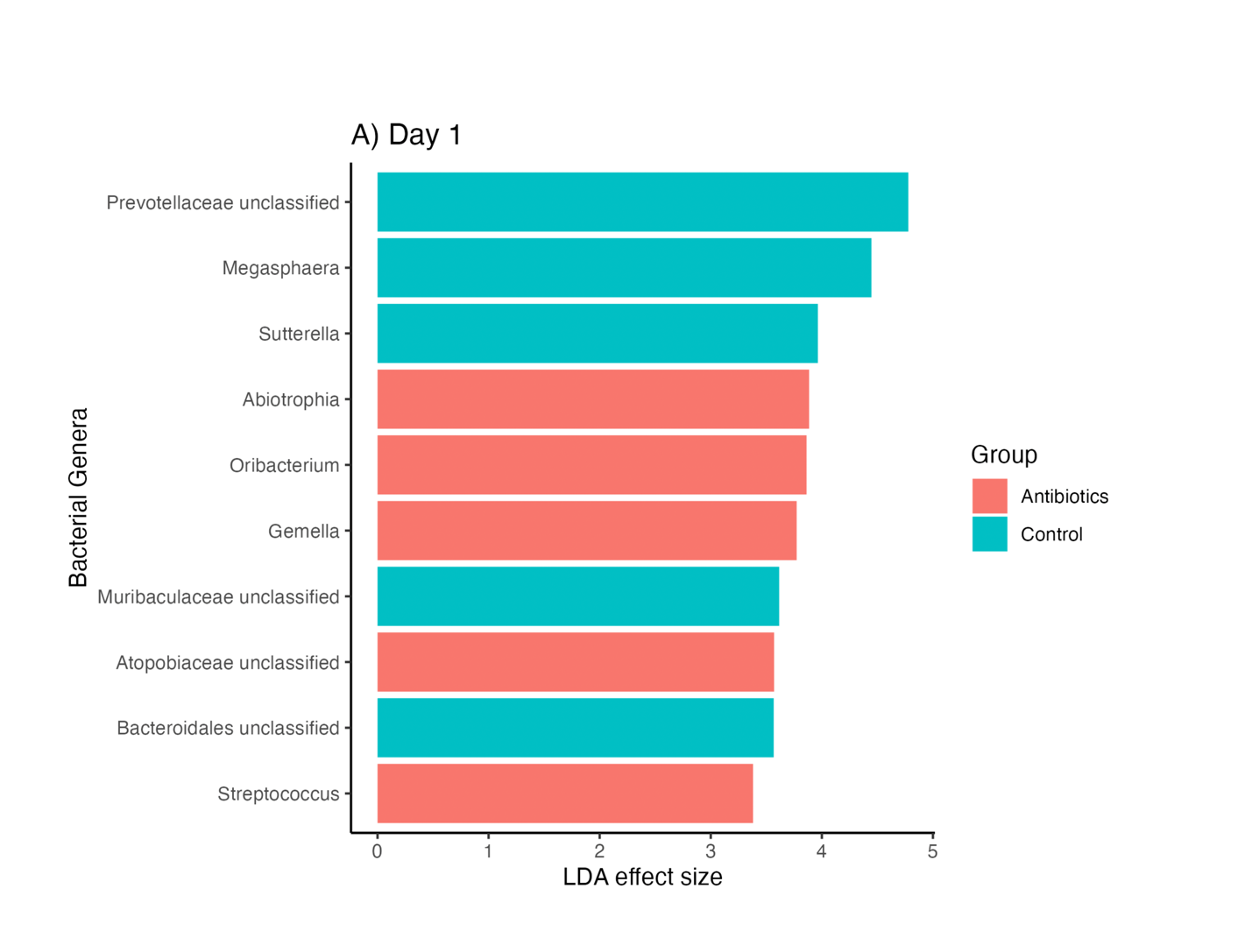


A) We assessed the taxa from the 16S rRNA data at the genus level to look for significant treatment effects of antibiotics using LEFSe. On the first day of the pre-treatment phase (day 1), LEFSe identified 10 bacterial genera that were differentially abundant between antibiotic and control groups. For reference, antibiotics were not administered until day 15 of this study, thus the microbiome data represented here is prior to antibiotic administration.


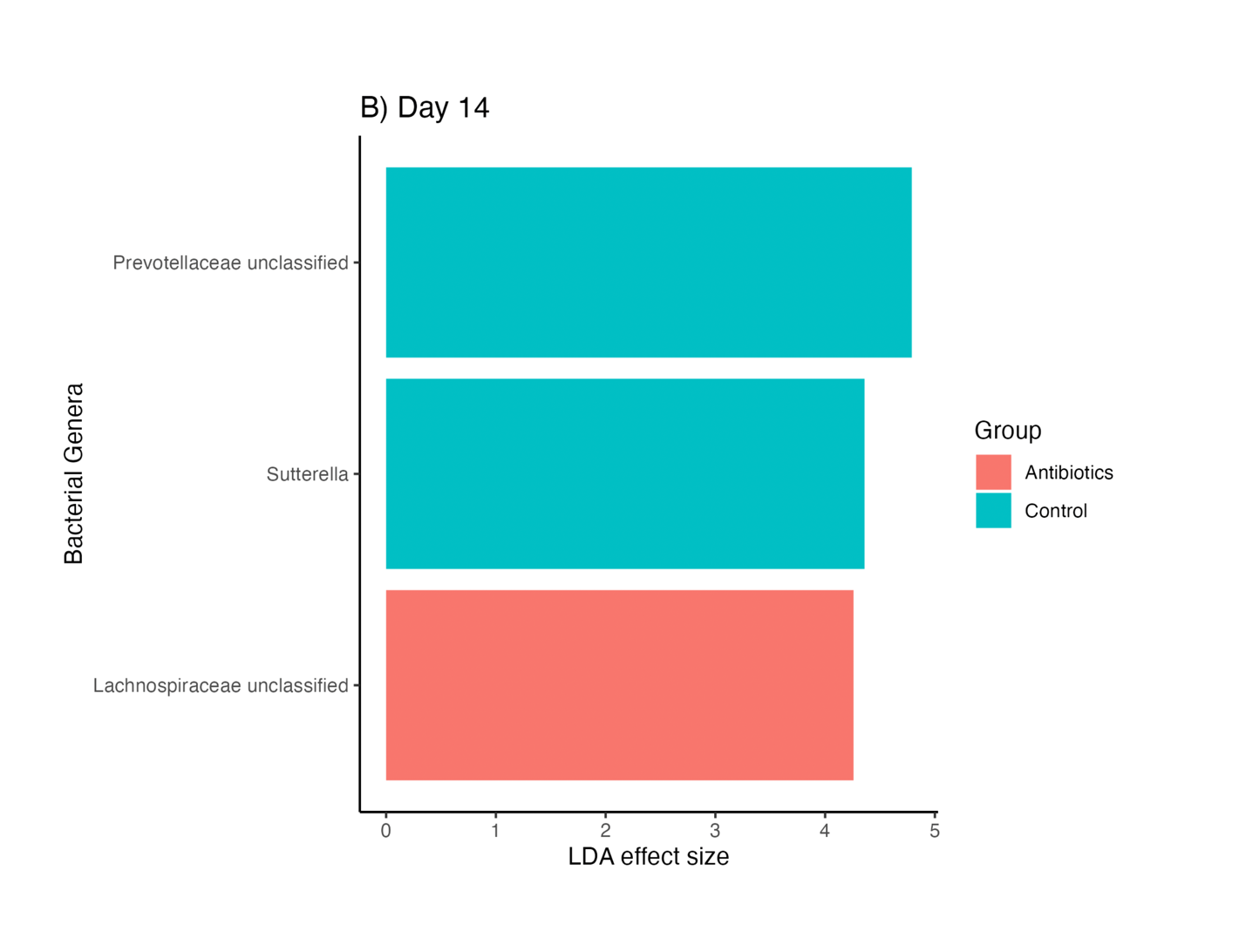


B) We assessed the taxa from the 16S rRNA data at the genus level to look for significant treatment effects of antibiotics using LEFSe. On the last day of the pre-treatment phase (day 14), LEFSe identified 3 bacterial genera that were differentially abundant between antibiotic and control groups. For reference, antibiotics were not administered until day 15 of this study, thus the microbiome data represented here is prior to antibiotic administration.
