# Supplementary material for: Antibiotic-induced gut dysbiosis elicits gut-brain axis relevant multi-omic signatures and behavioral and neuroendocrine changes in a nonhuman primate model
Source: Gut Microbes. 2024 Jan 29;16(1):2305476. doi: 10.1080/19490976.2024.2305476 (PMC10826635; doi:10.1080/19490976.2024.2305476)
Supplement: hayer_et_al_gut_microbes_supplementary_material_8.docx [file KGMI_A_2305476_SM1818.docx]

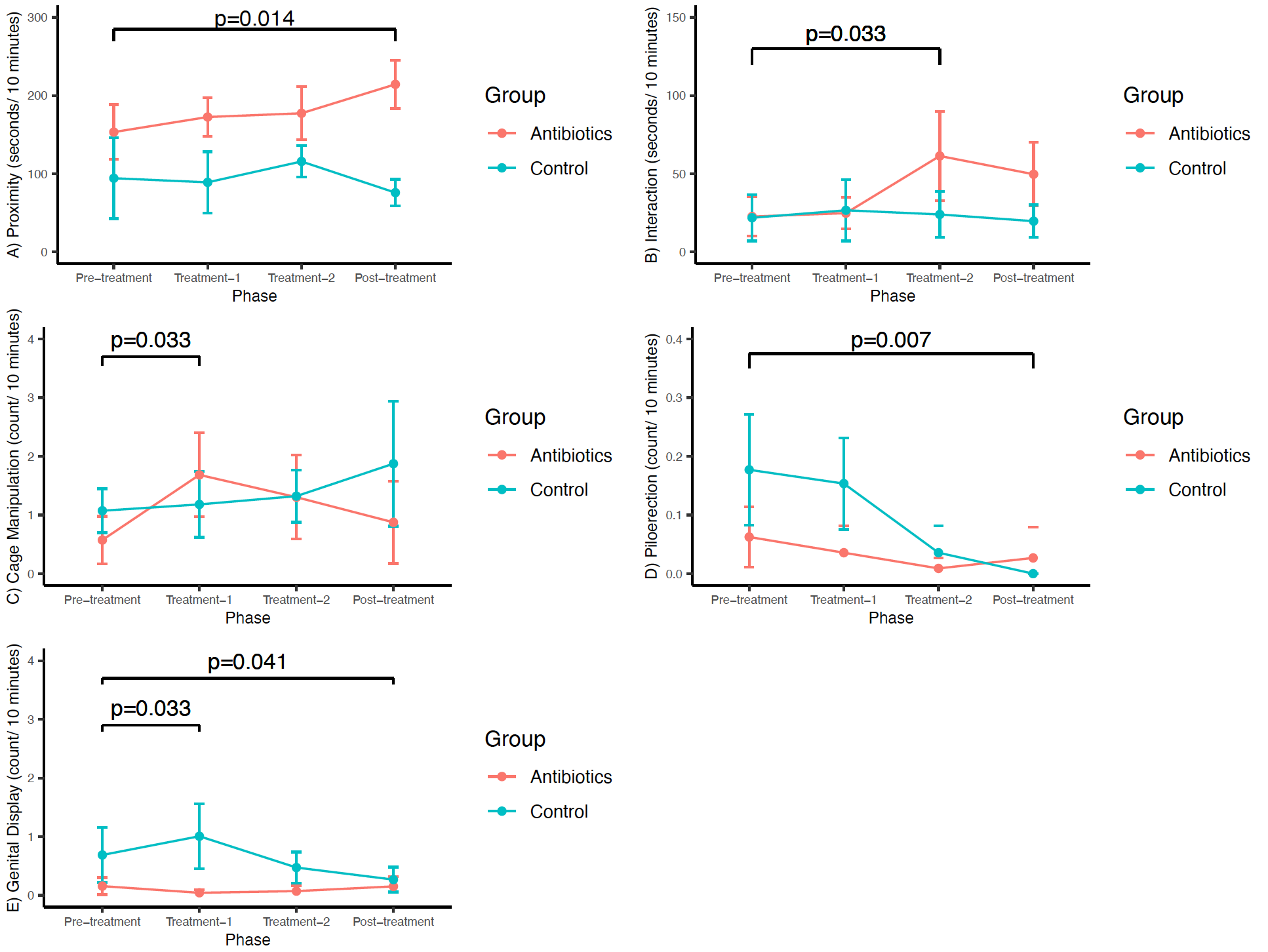


Changes in behavior during the course of the experiment (box-plots). p-values are highlighted whenever there is a significant group-time interaction (p <0.05) with relation to pre-treatment phase and control group (reference points) in mixed-effects models.
