# Supplementary material for: Antibiotic-induced gut dysbiosis elicits gut-brain axis relevant multi-omic signatures and behavioral and neuroendocrine changes in a nonhuman primate model
Source: Gut Microbes. 2024 Jan 29;16(1):2305476. doi: 10.1080/19490976.2024.2305476 (PMC10826635; doi:10.1080/19490976.2024.2305476)
Supplement: hayer_et_al_gut_microbes_supplementary_material_2_revision.docx [file KGMI_A_2305476_SM1815.docx]

QIIME 2: [Bolyen E, Rideout JR, Dillon MR, Bokulich NA, Abnet CC, Al-Ghalith GA, Alexander H, Alm EJ, Arumugam M, Asnicar F, et al. Reproducible, interactive, scalable and extensible microbiome data science using QIIME 2. Nat Biotechnol 2019; 37:852–7.](http://paperpile.com/b/iMxSs0/U2ZEE)

DADA2 pipeline: [Callahan BJ, McMurdie PJ, Rosen MJ, Han AW, Johnson AJA, Holmes SP. DADA2: High-resolution sample inference from Illumina amplicon data. Nat Methods 2016; 13:581–3.](http://paperpile.com/b/iMxSs0/LllRw)

SILVA database: [Quast C, Pruesse E, Yilmaz P, Gerken J, Schweer T, Yarza P, Peplies J, Glöckner FO. The SILVA ribosomal RNA gene database project: improved data processing and web-based tools. Nucleic Acids Res 2013; 41:D590–6.](http://paperpile.com/b/iMxSs0/x9iKe)

R studio: [Ihaka R, Gentleman R. R: A Language for Data Analysis and Graphics. J Comput Graph Stat 1996; 5:299–314.](http://paperpile.com/b/iMxSs0/aWcBk)

Trimmomatic: [Bolger AM, Lohse M, Usadel B. Trimmomatic: a flexible trimmer for Illumina sequence data. Bioinformatics 2014; 30:2114–20.](http://paperpile.com/b/iMxSs0/AxsoI)

FastQC: [Andrews S, Others. FastQC: a quality control tool for high throughput sequence data. 2010;](http://paperpile.com/b/iMxSs0/uguJO)

Bowtie2: [Langmead B, Salzberg SL. Fast gapped-read alignment with Bowtie 2. Nat Methods 2012; 9:357–9.](http://paperpile.com/b/iMxSs0/nnMk4)

MetaPhlan: [Blanco-Miguez A, Beghini F, Cumbo F, McIver LJ, Thompson KN, Zolfo M, Manghi P, Dubois L, Huang KD, Thomas AM, et al. Extending and improving metagenomic taxonomic profiling with uncharacterized species with MetaPhlAn 4 [Internet]. bioRxiv2022 [cited 2022 Oct 29]; :2022.08.22.504593. Available from:](http://paperpile.com/b/iMxSs0/Qob0l) <https://www.biorxiv.org/content/10.1101/2022.08.22.504593>

LefSe: [Segata N, Izard J, Waldron L, Gevers D, Miropolsky L, Garrett WS, Huttenhower C. Metagenomic biomarker discovery and explanation. Genome Biol 2011; 12:R60.](http://paperpile.com/b/iMxSs0/670hx)

Maaslin2: Mallick H, Rahnavard A, McIver LJ, Ma S, Zhang Y, Nguyen LH, Tickle TL, Weingart G, Ren B, Schwager EH, Chatterjee S, Thompson KN, Wilkinson JE, Subramanian A, Lu Y, Waldron L, Paulson JN, Franzosa EA, Bravo HC, Huttenhower C (2021). [[Multivariable Association Discovery in Population-scale Meta-omics Studies](https://journals.plos.org/ploscompbiol/article?id=10.1371/journal.pcbi.1009442)]. PLoS Computational Biology, 17(11):e1009442

MetaboAnalyst: [Pang Z, Chong J, Zhou G, de Lima Morais DA, Chang L, Barrette M, Gauthier C, Jacques P-É, Li S, Xia J. MetaboAnalyst 5.0: narrowing the gap between raw spectra and functional insights. Nucleic Acids Res 2021; 49:W388–96.](http://paperpile.com/b/iMxSs0/8RD0X)

False Discovery Rate: [Benjamini Y, Hochberg Y. Controlling the false discovery rate: A practical and powerful approach to multiple testing. J R Stat Soc 1995; 57:289–300.](http://paperpile.com/b/iMxSs0/UmYdY)
